# Estimating global and regional disruptions to routine childhood vaccine coverage during the COVID-19 pandemic in 2020: a modelling study

**DOI:** 10.1016/S0140-6736(21)01337-4

**Published:** 2021-08-07

**Authors:** Kate Causey, Nancy Fullman, Reed J D Sorensen, Natalie C Galles, Peng Zheng, Aleksandr Aravkin, M Carolina Danovaro-Holliday, Ramon Martinez-Piedra, Samir V Sodha, Martha Patricia Velandia-González, Marta Gacic-Dobo, Emma Castro, Jiawei He, Megan Schipp, Amanda Deen, Simon I Hay, Stephen S Lim, Jonathan F Mosser

**Affiliations:** a Institute for Health Metrics and Evaluation, University of Washington, Seattle, WA, USA; b Department of Health Metrics Sciences, School of Medicine, University of Washington, Seattle, WA, USA; c Department of Applied Mathematics, University of Washington, Seattle, WA, USA; d Department of Immunization, Vaccines and Biologicals, Word Health Organization, Geneva, Switzerland; e Pan American Health Organization, Comprehensive Family Immunization Unit, Washington, DC, USA; f Pediatric Infectious Diseases, Seattle Children's Hospital, Seattle, WA, USA

## Abstract

**Background:**

The COVID-19 pandemic and efforts to reduce SARS-CoV-2 transmission substantially affected health services worldwide. To better understand the impact of the pandemic on childhood routine immunisation, we estimated disruptions in vaccine coverage associated with the pandemic in 2020, globally and by Global Burden of Disease (GBD) super-region.

**Methods:**

For this analysis we used a two-step hierarchical random spline modelling approach to estimate global and regional disruptions to routine immunisation using administrative data and reports from electronic immunisation systems, with mobility data as a model input. Paired with estimates of vaccine coverage expected in the absence of COVID-19, which were derived from vaccine coverage models from GBD 2020, Release 1 (GBD 2020 R1), we estimated the number of children who missed routinely delivered doses of the third-dose diphtheria-tetanus-pertussis (DTP3) vaccine and first-dose measles-containing vaccine (MCV1) in 2020.

**Findings:**

Globally, in 2020, estimated vaccine coverage was 76·7% (95% uncertainty interval 74·3–78·6) for DTP3 and 78·9% (74·8–81·9) for MCV1, representing relative reductions of 7·7% (6·0–10·1) for DTP3 and 7·9% (5·2–11·7) for MCV1, compared to expected doses delivered in the absence of the COVID-19 pandemic. From January to December, 2020, we estimated that 30·0 million (27·6–33·1) children missed doses of DTP3 and 27·2 million (23·4–32·5) children missed MCV1 doses. Compared to expected gaps in coverage for eligible children in 2020, these estimates represented an additional 8·5 million (6·5–11·6) children not routinely vaccinated with DTP3 and an additional 8·9 million (5·7–13·7) children not routinely vaccinated with MCV1 attributable to the COVID-19 pandemic. Globally, monthly disruptions were highest in April, 2020, across all GBD super-regions, with 4·6 million (4·0–5·4) children missing doses of DTP3 and 4·4 million (3·7–5·2) children missing doses of MCV1. Every GBD super-region saw reductions in vaccine coverage in March and April, with the most severe annual impacts in north Africa and the Middle East, south Asia, and Latin America and the Caribbean. We estimated the lowest annual reductions in vaccine delivery in sub-Saharan Africa, where disruptions remained minimal throughout the year. For some super-regions, including southeast Asia, east Asia, and Oceania for both DTP3 and MCV1, the high-income super-region for DTP3, and south Asia for MCV1, estimates suggest that monthly doses were delivered at or above expected levels during the second half of 2020.

**Interpretation:**

Routine immunisation services faced stark challenges in 2020, with the COVID-19 pandemic causing the most widespread and largest global disruption in recent history. Although the latest coverage trajectories point towards recovery in some regions, a combination of lagging catch-up immunisation services, continued SARS-CoV-2 transmission, and persistent gaps in vaccine coverage before the pandemic still left millions of children under-vaccinated or unvaccinated against preventable diseases at the end of 2020, and these gaps are likely to extend throughout 2021. Strengthening routine immunisation data systems and efforts to target resources and outreach will be essential to minimise the risk of vaccine-preventable disease outbreaks, reach children who missed routine vaccine doses during the pandemic, and accelerate progress towards higher and more equitable vaccination coverage over the next decade.

**Funding:**

Bill & Melinda Gates Foundation.

## Introduction

SARS-CoV-2, the virus responsible for COVID-19, rapidly evolved from a localised outbreak in December, 2019, into a pandemic responsible for more than 79 million confirmed cases and 1·7 million deaths worldwide by the end of 2020.^1^ Health services were substantially affected by the COVID-19 crisis^2^, ^3^ because of the restrictions placed on movement and travel, health facility capacity (eg, health workers were being deployed to COVID-19 wards and there was inadequate supply of personal protective equipment [PPE]) or demand (eg, patients had non-urgent medical care postponed because of concerns about exposure to the virus), or a combination of these factors.^4^, ^5^, ^6^ According to a WHO report published in August, 2020,^7^ 90% of 105 countries reported at least some disruptions to essential health services, with routine immunisation services among the most frequently disrupted. With global gains in childhood vaccine coverage stalling in recent years and most locations already falling short of 90% global coverage targets in 2019,^8^ any additional declines in vaccination rates pose massive risks to child health and survival.^9^

**Research in context**

**Evidence before this study**

As SARS-CoV-2 rapidly spread worldwide in early 2020 and governments sought to curb transmission, many health services, especially routine immunisation, faced severe disruptions. Such effects stemmed from numerous factors, including travel restrictions and policies aiming to reduce person-to-person contact and social mixing, deployment of health workers for the COVID-19 response, and cancelled or postponed patient visits because of concerns of viral exposure, among others. During the earlier phases of the COVID-19 pandemic, agencies and organisations including WHO, UNICEF, and Gavi, the Vaccine Alliance collected qualitative information from country experts about disruptions to routine immunisation programmes. These data, published as pulse polls and interim reports, shed light on the initial magnitude of disruptions to routine immunisation services, with 126 (74%) of 170 countries reporting at least some disruption. Increasingly, more studies have sought to quantify the impact of the pandemic on routine immunisation via administrative data sources, and have shown the acute effects of the pandemic on vaccine doses delivered and vaccine-preventable diseases; however, these studies have generally been limited either to specific locations or to certain time periods, or both. Some modelling studies have considered different scenarios for the disruption to routine immunisation services, estimating the potential effects of pre-specified reductions in vaccine coverage on disease burden. However, to date, no modelled analysis has sought to comprehensively estimate disruptions to routine immunisation at global and regional levels throughout 2020.

**Added value of this study**

In this analysis, we assess global and regional patterns in disruptions to routine immunisation attributable to the COVID-19 pandemic in 2020. Drawing from country-reported data and supplementary sources, we estimated monthly disruptions in administration of the diphtheria-tetanus-pertussis, third dose (DTP3) vaccine and measles-containing vaccine, first dose (MCV1). These estimates were based on a two-step model, in which cascading splines were fit to data on the number of vaccines administered. The model used mobility measures as a predictor of the effects of COVID-19 on human movement and interactions, but also allowed for the pace of disruption and recovery in vaccination to differ from mobility trends where suggested by the data. Using vaccine coverage data from 1980 to 2019 and models of vaccine coverage from the Global Burden of Diseases, Injuries, and Risk Factors Study (GBD) 2020, Release 1 (GBD 2020 R1), we estimated expected DTP3 and MCV1 coverage for 2020 in the absence of the COVID-19 pandemic. We used these expected values to quantify disruptions to routine immunisation that were attributable to COVID-19, including the additional number of children who missed doses of DTP3 and MCV1 throughout 2020. This analysis offers, to our knowledge, the first modelled assessments of global and regional disruptions to vaccination coverage, by month, during the COVID pandemic in 2020.

**Implications of all the available evidence**

Globally, marked disruptions to routine immunisation services occurred in 2020, with DTP3 and MCV1 coverage estimated to have fallen more than 7% worldwide compared to expected coverage in the absence of COVID-19. For both DTP3 and MCV1, more than 8 million additional children missed doses beyond expected estimates of vaccination gaps for 2020, underscoring the magnitude of COVID-19-related disruptions to routine immunisation and the potential risk of future infectious disease outbreaks. Gradual but steady recovery appears to be underway, but several factors—ongoing transmission, emergence of new variants, and a focus on the roll-out of COVID-19 vaccines, among others—could easily stall or reverse these trends. In tandem, routine immunisation data systems must be strengthened to enable data-informed decision making at local levels and improved monitoring of routine immunisation over time.

Recent analyses suggest that various countries experienced disruptions to routine immunisation programmes or corresponding decreases in vaccine coverage, or both, in 2020, especially during the earlier phases of the COVID-19 pandemic.^4^, ^6^, ^10^, ^11^, ^12^, ^13^ According to a poll of 260 health-care practitioners in May, 2020, respondents in 53 (85%) of 61 countries reported lower vaccination levels than those recorded in January and February, 2020.^4^ A systematic review of 17 observational studies found consistent declines in vaccine coverage and administered doses across locations and over time.^6^ Additionally, the twenty-eighth meeting of the Emergency Committee under the International Health Regulations noted that ongoing COVID-19 transmission continues to pose a risk to polio eradication efforts, both for wild-type polio eradication activities and control of circulating vaccine-derived polioviruses.^14^ Analyses across 21 countries in Europe, sub-Saharan Africa, north Africa and the Middle East, and south Asia during the first half of 2020 found disruptions to childhood vaccination programmes of up to 90%.^10^, ^11^, ^12^, ^13^, ^15^ These studies, however, have been limited to a subset of locations and time periods earlier in the pandemic. Furthermore, such work has generally focused solely on administrative data or modelling hypothetical disruption scenarios. Although both of these study types can provide insights into the effects of the COVID-19 pandemic on routine immunisation, they can be prone to reporting issues and might not fully reflect how COVID-19 has affected routine immunisation services across different regions. Generating comparable global and regional estimates of disruptions to routine immunisation services throughout 2020 is crucial to our understanding of routine immunisation recovery and expansion needs in both the immediate future and beyond the COVID-19 pandemic.

In this analysis, we aimed to quantify the effects of the COVID-19 pandemic on routine immunisation through to December, 2020. First, we collated country-reported data on the monthly number of doses delivered for the third dose of a diphtheria-tetanus-pertussis vaccine (DTP3) and the first dose of a measles-containing vaccine (MCV1) between January, 2019, and December, 2020.^16^ We then supplemented these data with reports based on electronic medical records and registries, as well as human mobility measures, to estimate short-term effects of the COVID-19 pandemic on monthly DTP3 and MCV1 coverage in 2020. Last, we compared these coverage estimates to those expected in 2020 in the absence of the COVID-19 pandemic, as derived from the Global Burden of Diseases, Injuries, and Risk Factors study (GBD) 2020, Release 1 (GBD 2020 R1) vaccine coverage models,^8^ and estimated the number of additional children who missed doses attributable to the pandemic. This study provides the first modelled estimates of the immediate effects of the COVID-19 pandemic on DTP3 and MCV1 coverage, by month in 2020, offering a data-driven platform to help inform near-term recovery and longer-term routine immunisation programme expansion so that all children can benefit from vaccines.

## Methods

### Overview

Our analysis involved three main steps: first, synthesising available administrative data and electronic records with a two-step model of monthly DTP3 and MCV1 disruptions in 2020, using human mobility data to inform these trends; second, quantifying disruptions attributable to COVID-19 on the basis of expected 2020 coverage levels; and third, calculating the additional number of children missing DTP3 and MCV1 doses over the course of 2020. Each step is summarised below and further detailed in the appendix (section 4.1).

This analysis complies with the Guidelines for Accurate and Transparent Health Estimates Reporting (GATHER) statement,^17^ with further information provided in the appendix (section 1); all source codes used to generate estimates and corresponding data will be made accessible upon publication, on the Global Health Data Exchange website.

### Data

We first collated total monthly DTP3 and MCV1 doses administered in both 2019 and 2020, as reported by countries to regional WHO offices. For countries with available data from both years for the baseline period of January and February, the number of paired-year country reports were highest in March (n=98) and April (n=97), then May (n=93), June (n=92), July (n=77), August (n=76), September (n=73), October (n=45), November (n=41), and December (n=15); cumulatively, these data represented nearly 230 million doses administered across 1806 location-month-years.^16^

We supplemented these administrative data by searching the published literature, ministry of health websites, and media reports. We included all dose or coverage data representative at the national level or first administrative unit for children aged 0–59 months; monthly patterns in 2019 and 2020 to capture trends in eligible populations and corresponding coverage; and data on three-dose coverage of any diphtheria-containing, tetanus-containing, and pertussis-containing vaccine (ie, DTP, pentavalent, or hexavalent combination vaccines) for the DTP3 model and one-dose coverage of any measles-containing vaccine (ie, measles-only, measles-rubella, or measles-mumps-rubella) for the MCV1 model. We excluded data where the full month of March, 2020, was not covered. More details of data processing are provided in the appendix (section 2).

**Figure 1:**
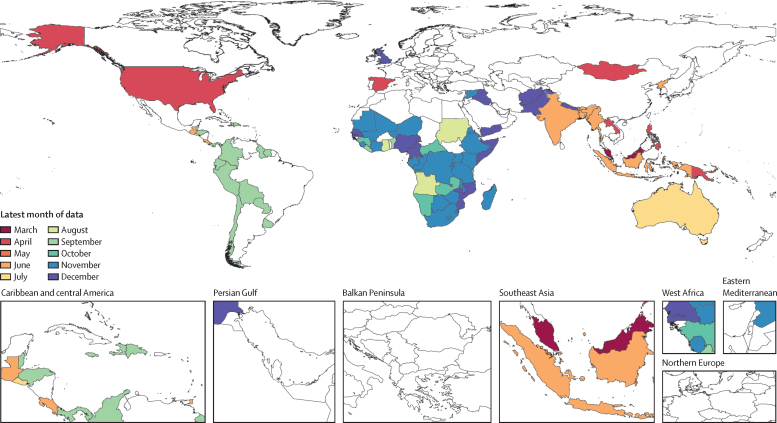
Latest month of vaccine data availability in 2020 Data on either vaccine (DTP3 or MCV1) are shown. Locations coloured white did not have available month-specific data on MCV1 or DTP3 for 2020 at the time of analysis. The boundaries and names shown and the designations used on this map do not imply the expression of any opinion whatsoever on the part of WHO concerning the legal status of any country, territory, city or area or of its authorities or concerning the delimitation of its frontiers or boundaries. Dotted and dashed lines on maps represent approximate border lines for which there may not yet be full agreement. DTP3=diphtheria-tetanus-pertussis, third dose. MCV1=measles-containing vaccine, first dose.

Additional vaccine delivery data were extracted from government-based health data tools or publications from Nigeria,^18^ Scotland,^19^ England,^20^ Australia,^21^ Spain,^22^ India,^23^ and Nepal,^24^ and media reports from the USA;^25^, ^26^ all data inputs are detailed in the appendix (table S1). Among 204 countries and territories, 94 (46%) had sufficient data for inclusion in this analysis: monthly data from at least March, 2020, and a suitable pre-pandemic reference period (figure 1, appendix table S1). Additional details about data availability by GBD super-region and the use of subnational data are available in the appendix (sections 2.1, 2.4, and 2.5).

By vaccine, we calculated each location's cumulative disruption ratio (*CDR*) from March to each month, *m*, relative to ratios of doses administered, *D*, in 2019 and 2020:

$$CDR_{m}=\frac{\left(\frac{D_{Marchtom,2020}}{D_{Marchtom,2019}}\right)}{\left(\frac{D_{JantoFeb,2020}}{D_{JantoFeb,2019}}\right)}$$

A monthly CDR of zero would indicate complete disruption of vaccine delivery between March, 2020, and the month in question, whereas a ratio of one indicates that the expected number of doses had been delivered during that period.

For location-months with CDRs for only one vaccine (DTP3 only: n=68; MCV1 only: n=5), we estimated values for the missing vaccine based on the average disruption ratio of MCV1 to DTP3, as weighted by the sum of the inverse variance of both ratios—an established strategy for meta-regression (appendix section 2.2).^27^

We used qualitative data collected by WHO and partners, including two sets of Pulse poll results,^4^, ^28^ essential health services polls,^7^ and additional data from WHO regional offices, to evaluate estimated CDRs. If discrepancies occurred between administrative data reports and qualitative sources, we reviewed administrative data reports for potential anomalies such as stockouts occurring in January to February, 2020, which would affect baseline values, and excluded those CDRs (168 of 1402 CDRs were thus excluded; appendix section 2.1). To account for potential reporting delays, we excluded any location's most recent month of data if there was more than a 20% decrease between the 2020 to 2019 ratio for the most recent month of data and the corresponding ratio for the previous month; this approach resulted in the exclusion of 25 of 1402 CDRs (appendix section 2.1). We also tested the sensitivity of the model to this assumption (appendix section 4.5). Finally, we excluded CDRs from countries for which we had an alternative source of administrative data (30 of 1402 CDRs were thus excluded; appendix section 2.1).

In the absence of more granular data on proximal determinants of vaccine coverage^29^ or specific types of disruptions during the COVID-19 pandemic, or both, we used human mobility estimates to inform our model of the time-varying effects of the pandemic on routine immunisation. Changes in mobility patterns compared to pre-pandemic levels reflect how people changed behaviours during the course of the COVID-19 pandemic and can thus serve as a time-varying partial proxy for broader societal disruptions related to the pandemic.^30^ Full methodological details about generating mobility estimates have been published elsewhere,^31^ but in brief, we synthesised daily human movement data obtained from mobile phones from Descartes Labs,^32^ Facebook,^33^ Google,^34^ and SafeGraph^35^ in 134 countries and territories into cohesive estimates of the relative decline in mobility compared to a pre-COVID-19 baseline. As such, cumulative mobility estimates were calculated as the average percentage reduction in mobility on a scale from 0 (no reduction in mobility) to 1 (100% reduction in mobility) from March 1, 2020, to that date. Averaged estimates by GBD region were used for the 70 countries for which data were not available (appendix section 2.5).

### Estimating disruptions to routine immunisation attributable to COVID-19

We used a two-step approach to estimate disruptions to DTP3 and MCV1 coverage attributable to the COVID-19 pandemic in 2020 (appendix section 4.1). In step 1, we modelled the average relationships between cumulative disruptions in human mobility and vaccine coverage. In step 2, we modelled the residual variation in disruption by month, further accounting for trends in disruption not explained by mobility alone. For each location, month, and vaccine, we combined results from the step 1 and step 2 models to produce trends in disruption estimates throughout 2020. Other covariates were considered in addition to our mobility measure (eg, total COVID-19 deaths and cases to date, indices of development,^36^ and health-system performance^37^), but they provided no meaningful associations with disruptions to vaccine coverage.

**Figure 2:**
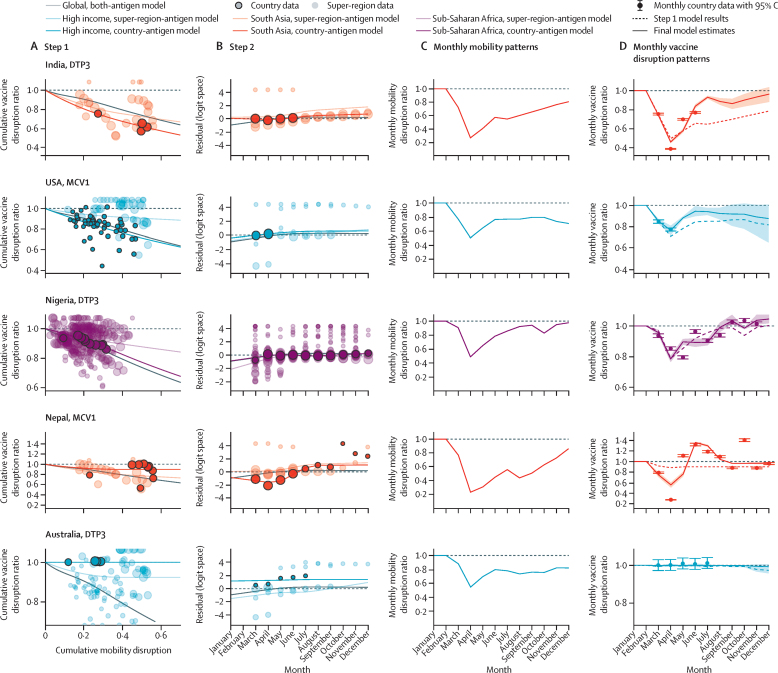
Examples of model performance for step 1 (A) and step 2 (B), monthly mobility patterns (C), and monthly vaccine disruption patterns (D), from January to December, 2020 Step 1 involved modelling the relationship between cumulative disruptions in mobility and vaccine coverage (A), and step 2 involved modelling residual variation (B); more detail is available in the main text and in the appendix (section 4.1). Global model estimates are represented by solid grey lines; GBD super-region data and antigen-specific estimates are represented by light coloured data points and lines; and location-specific and antigen-specific data and estimates are represented by dark coloured data points and lines. The size of each data point corresponds with the inverse variance and weight in the model. In panel C, mean estimates of monthly mobility patterns are shown by solid lines. In panel D, mean estimates from step 1 are represented by dashed coloured lines and final combined model estimates are represented by solid lines; shaded areas represent 95% UIs for the final combined model, and error bars represent the 95% CIs for the monthly country data. Location-specific and antigen-specific examples were selected to reflect a range of model fits across different patterns of mobility and vaccine disruption, residual trends, and data availability. GBD=Global Burden of Diseases, Injuries, and Risk Factors Study. DTP3=diphtheria-tetanus-pertussis, third dose. MCV1=measles-containing vaccine, first dose. UI=uncertainty interval.

For both steps, we used a constrained Bayesian meta-regression tool^38^ to fit a cascading random spline model (appendix section 3). This approach borrows strength across geographies and vaccines, while allowing individual locations to differ from global or super-regional trends where supported by data. We fit one global spline including data from both vaccines, seven by GBD super-region, 14 by GBD super-region and vaccine, and 188 location-specific and vaccine-specific splines, with each further disaggregated model borrowing strength from the previous one in the hierarchy (appendix section 3.4). We constrained estimated CDRs between 0 and 1 such that no country could exceed the expected 2020 vaccine coverage for the entire year; however, the monthly disruption ratio (MDR) could exceed 1 (eg, in the case of successful catch-up vaccination efforts) if supported by available data. Figure 2 provides model results for five countries, each representing different data availability and disruption trajectories in 2020. For locations without data, we estimated disruptions on the basis of GBD-super-region-specific and vaccine-specific splines and location-specific mobility data. For central Europe, eastern Europe, and central Asia, we estimated disruptions on the basis of global splines for steps 1 and 2 because of limited country data (n=1). Additional details of modelling procedures and uncertainty estimation are available in the appendix (sections 3.5 and 4.2). Briefly, we generated 1000 sets of estimates for both steps using asymptotic statistics and a log-normal distribution to generate mean estimates and 95% uncertainty intervals (UIs).

Model fit was evaluated on the basis of average disruption ratios computed for each location-vaccine-data source compared to modelled estimates of these ratios. We evaluated bias and goodness of fit for each modelling step by vaccine, weighting by the inverse variance of the log ratio (appendix section 4.3). The final combined model showed good fit to the data (appendix section 4.3), with weighted mean errors of −1·11% for DTP3 and −1·67% for MCV1, and weighted mean absolute errors of 1·31% for DTP3 and 1·80% for MCV1. We also did two out-of-sample validity analyses, described in the appendix (section 4.4).

### Estimating monthly number of children missing vaccine doses

Drawing from GBD 2020 R1 coverage estimates for DTP3 and MCV1,^8^ we estimated expected 2020 coverage levels in the absence of COVID-19. More detail is provided in the appendix (sections 4.6 and 4.7); in brief, we fit statistical models to available coverage data between 1980 and 2019, then predicted coverage values in 2020 assuming past trends in coverage and covariates would have continued in the absence of the pandemic.

To estimate monthly (*m*) missed doses, *N*, we converted estimated *CDRs* to *MDRs* and then combined them with expected vaccine coverage, *C*, and the estimated monthly number of eligible children calculated from GBD 2020 R1 annual target population estimates (updated from GBD 2019^36^ as part of the GBD continuous update cycle), *pop*_m_:

$$N_{m}=(1-MDR_{m}\times C)\times pop_{m}$$

1000 draws of *N* were calculated from draws of *MDR* and *C* to incorporate uncertainty in both quantities. For DTP3 we used 2020 population estimates for children younger than 1 year of age (ie, <12 months), and for MCV1 we used either this value (n=79) or population estimates for children aged 12–23 months (n=125) on the basis of the location's MCV1 schedule.^39^ We then aggregated these estimates to global and GBD super-region levels.

### Role of the funding source

The funder of the study had no role in study design, data collection, data analysis, data interpretation, or writing of the report.

## Results

**Figure 3:**
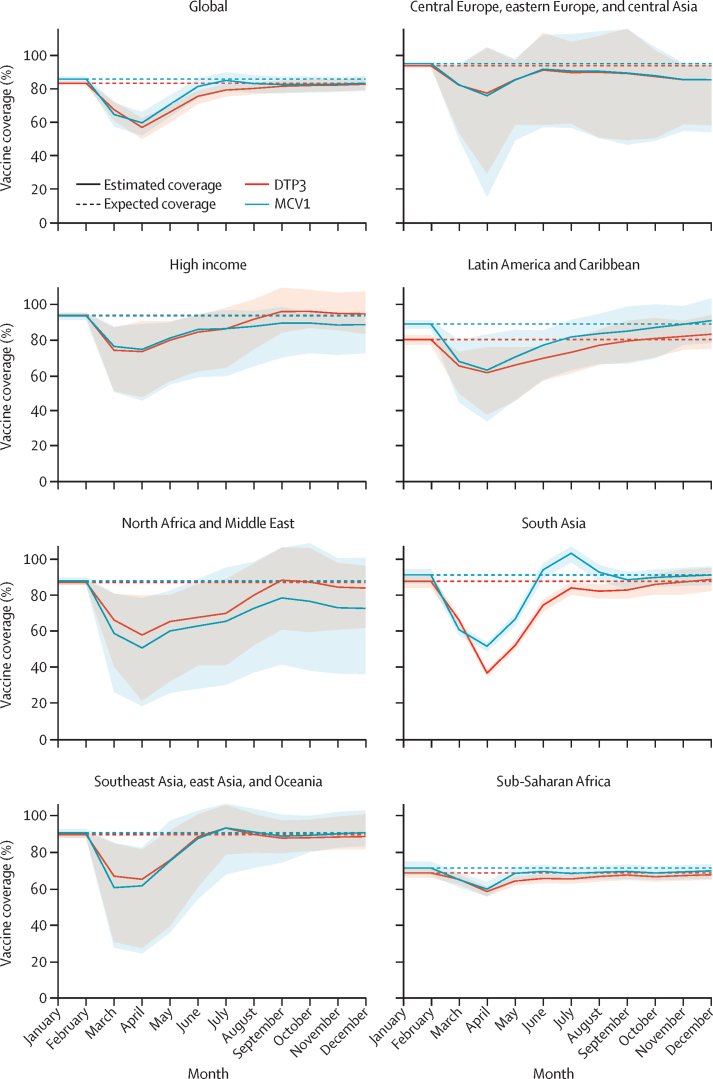
Monthly vaccine coverage for DTP3 and MCV1, globally and by GBD super-region, from January to December, 2020 Solid lines show the estimated coverage by vaccine for each GBD super-region and globally by month, and the shaded area displays the 95% uncertainty interval. Dashed lines display the expected coverage for each vaccine in the absence of COVID-19. Estimated coverage can temporarily exceed 100% because of catch-up of children missed in earlier months. DTP3=diphtheria-tetanus-pertussis, third dose. MCV1=measles-containing vaccine, first dose. GBD=Global Burden of Diseases, Injuries, and Risk Factors Study.

Globally, DTP3 coverage estimates for 2020 were 7·7% (95% UI 6·0 to 10·1) lower than expected and MCV1 coverage estimates were 7·9% (5·2 to 11·7) lower than expected in the absence of COVID-19 (figure 3, table). Disruptions were most severe in April, 2020, across all GBD super-regions, with the global number of doses administered falling by 31·3% (25·4 to 38·9) for DTP3 and by 30·1% (23·2 to 37·9) for MCV1 compared to expected levels. Between May and December, 2020, however, vaccine delivery appeared to improve, with the monthly estimated doses administered approaching expected levels by the end of the year (ie, estimated relative changes of −0·7% [–4·0 to 2·9] for DTP3 and −3·0% [–7·5 to 1·2] for MCV1 in December, 2020, compared to expected levels).

**Table:** Expected DTP3 and MCV1 coverage and missed doses in the absence of COVID-19 and estimates of disruptions, coverage, and doses missed attributable to the COVID-19 pandemic in 2020, globally, and by GBD super-region

	**DTP3**						**MCV1**					
	Expected coverage in the absence of COVID-19 (95% UI)	Estimated relative disruption attributable to COVID-19 (95% UI)	Estimated coverage after accounting for COVID-19 disruption (95% UI)	Expected doses missed in the absence of COVID-19, millions (95% UI)	Estimated doses missed attributable to COVID-19, millions (95% UI)	Estimated total doses missed after accounting for COVID-19, millions (95% UI)	Expected coverage in the absence of COVID-19 (95% UI)	Estimated relative disruption attributable to COVID-19 (95% UI)	Estimated coverage after accounting for COVID-19 disruption (95% UI)	Expected doses missed in the absence of COVID-19, millions (95% UI)	Estimated doses missed attributable to COVID-19, millions (95% UI)	Estimated total doses missed after accounting for COVID-19, millions (95% UI)
Global	83·3% (82·1–84·4)	7·7% (6·0–10·1)	76·7% (74·3–78·6)	21·5 (20·0–23·0)	8·5 (6·5–11·6)	30·0 (27·6–33·1)	85·9% (84·3–87·4)	7·9% (5·2–11·7)	78·9% (74·8–81·9)	18·2 (16·3–20·3)	8·9 (5·7–13·7)	27·2 (23·4–32·5)
Central Europe, eastern Europe, and central Asia	93·8% (92·7–94·6)	6·7% (0·1–28·8)	87·6% (66·5–94·2)	0·3 (0·3–0·4)	0·3 (0·0–1·4)	0·7 (0·3–1·8)	95·1% (94·1–95·9)	7·6% (0·1–32·8)	87·9% (63·9–95·5)	0·3 (0·2–0·3)	0·4 (0·0–1·7)	0·7 (0·2–1·9)
High income	94·3% (93·2–95·4)	6·1% (2·2–16·8)	88·6% (78·5–92·4)	0·6 (0·5–0·7)	0·6 (0·2–1·7)	1·2 (0·8–2·3)	94·0% (91·7–95·8)	7·9% (2·8–27·0)	86·5% (68·3–92·1)	0·7 (0·5–0·9)	0·8 (0·3–2·8)	1·5 (0·9–3·5)
Latin America and Caribbean	80·4% (77·4–82·9)	6·6% (3·9–14·0)	75·0% (68·8–78·9)	1·8 (1·6–2·1)	0·5 (0·3–1·0)	2·3 (2·0–2·9)	89·3% (86·6–91·4)	9·2% (4·6–21·9)	81·3% (68·6–86·7)	1·0 (0·8–1·3)	0·7 (0·4–1·8)	1·8 (1·2–2·9)
North Africa and Middle East	87·4% (86·0–88·8)	11·0% (2·0–30·4)	77·4% (58·8–86·5)	1·5 (1·3–1·7)	1·2 (0·2–3·4)	2·7 (1·6–4·9)	88·3% (86·0–90·3)	18·9% (3·0–49·1)	70·8% (42·3–86·6)	1·4 (1·2–1·7)	2·1 (0·3–5·6)	3·5 (1·6–7·0)
South Asia	88·0% (84·2–91·1)	13·0% (11·0–15·1)	76·6% (72·9–79·9)	3·7 (2·8–4·9)	3·6 (3·0–4·2)	7·3 (6·3–8·5)	91·6% (86·9–94·8)	7·4% (6·1–8·7)	84·6% (80·2–88·0)	2·6 (1·6–4·1)	2·2 (1·8–2·6)	4·8 (3·8–6·2)
Southeast Asia, east Asia, and Oceania	89·9% (88·1–91·4)	6·4% (3·4–18·0)	84·4% (72·9–87·8)	2·5 (2·1–3·0)	1·4 (0·7–4·1)	3·9 (3·1–6·8)	90·8% (88·1–93·0)	7·5% (3·6–25·1)	84·3% (67·6–88·7)	2·3 (1·8–3·0)	1·6 (0·7–5·8)	3·9 (2·8–8·1)
Sub-Saharan Africa	68·7% (66·2–71·2)	3·8% (3·4–4·8)	66·1% (63·5–68·4)	10·9 (10·1–11·8)	0·9 (0·8–1·2)	11·8 (11·0–12·7)	71·5% (67·5–75·0)	4·4% (3·7–5·9)	68·4% (64·6–71·8)	9·9 (8·7–11·3)	1·1 (0·9–1·5)	11·0 (9·8–12·3)

Expected coverage corresponds to expected levels for 2020 in the absence of the COVID-19 pandemic, based on past trends. Estimated coverage reflects coverage for 2020 while accounting for estimated disruptions related to the COVID-19 pandemic; more detail about these methods is provided in the main paper and appendix. Doses missed reflect the number of children who were eligible to receive a given vaccine at some point during the year (ie, the target population) but did not receive it by the end of 2020. DTP3=diphtheria-tetanus-pertussis, third dose. MCV1=measles-containing vaccine, first dose. UI=uncertainty interval. GBD=Global Burden of Diseases, Injuries, and Risk Factors Study.

Among all GBD super-regions (figure 3, table), south Asia experienced the largest acute declines, with DTP3 doses administered falling by 58·3% (95% UI 57·4–59·4) and MCV1 doses falling by 43·1% (42·1–44·1) in April, 2020, compared to expected levels. Since then, improvements occurred, with south Asia nearing expected levels of monthly administration of DTP3 and MCV1 by the end of 2020. Nonetheless, annual vaccine delivery still fell short, with reductions of 13·0% (11·0–15·1) in DTP3 and 7·4% (6·1–8·7) in MCV1, compared to expected levels of administration for 2020. By contrast, for north Africa and the Middle East, where disruptions were less acute but recovery seems to have plateaued, the yearly numbers of administered doses were 11·0% (2·0–30·4) lower than expected values for DTP3 and 18·9% (3·0–49·1) lower than expected values for MCV1. Sub-Saharan Africa had smaller acute disruptions in doses administered during this time, with estimated annual DTP3 doses administered being 3·8% (3·4–4·8) lower and estimated annual MCV1 doses being 4·4% (3·7–5·9) lower than expected for 2020. These patterns might be related to the comparatively smaller scale of the COVID-19 pandemic in this GBD super-region,^1^, ^40^ particularly during the first half of 2020. Conversely, many countries in the high-income super-region, including the USA, have had among the world's largest COVID-19 outbreaks.^1^, ^40^ For the high-income super-region, estimated DTP3 doses administered fell by 22·1% (3·8–49·2) and MCV1 doses fell by 20·5% (5·0–50·8) in April, 2020, the super-region's nadir compared to expected values. Partial recovery then occurred through to December, 2020, with annual reductions of 6·1% (2·2–16·8) for DTP3 and 7·9% (2·8–27·0) for MCV1, compared with expected levels of dose administration.

In the absence of the COVID-19 pandemic, expected global coverage in 2020 was predicted to be 83·3% (95% UI 82·1–84·4) for DTP3 and 85·9% (84·3–87·4) for MCV1. After accounting for pandemic-associated disruptions, however, estimated coverage in 2020 was 76·7% (74·3–78·6) for DTP3 and 78·9% (74·8–81·9) for MCV1. In north Africa and the Middle East, expected 2020 coverage levels exceeded 85% (ie, 87·4% [86·0–88·8] for DTP3 and 88·3% [86·0–90·3] for MCV1) if the pandemic had not occurred, whereas estimated 2020 coverage accounting for COVID-19-related disruptions was 10–20 percentage points lower (ie, 77·4% [58·8–86·5] for DTP3 and 70·8% [42·3–86·6] for MCV1). South Asia experienced the largest gap between expected and estimated 2020 coverage for DTP3: expected coverage was 88·0% (84·2–91·1) for 2020, but after accounting for pandemic-related disruptions, estimated DTP3 coverage was 76·6% (72·9–79·9). Sub-Saharan Africa had the smallest absolute declines in coverage attributable to the COVID-19 pandemic. Because of pre-pandemic trends, however, the estimated coverage for 2020 in sub-Saharan Africa was the lowest across all GBD super-regions: 66·1% (63·5–68·4) for DTP3 and 68·4% (64·6–71·8) for MCV1. Estimates by country are provided in the appendix (table S2).

**Figure 4:**
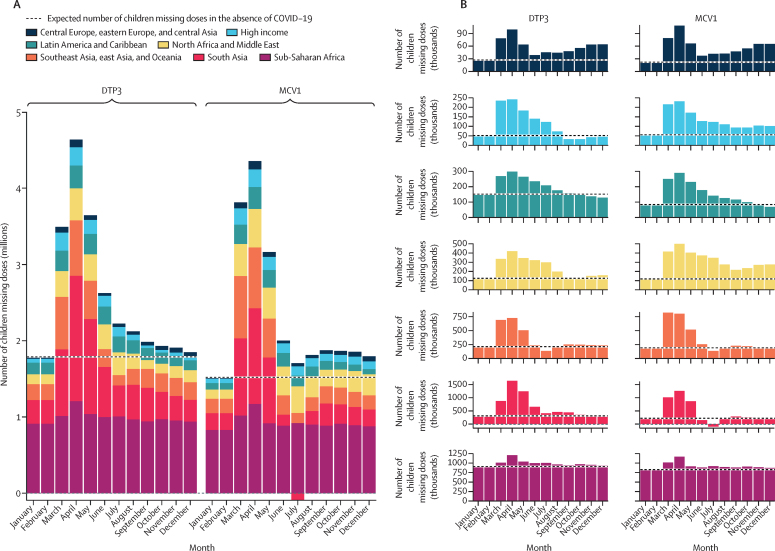
Estimated number of eligible children missing doses of DTP3 and MCV1 globally (A), and by GBD super-region (B), from January to December, 2020 The expected number of eligible children missing doses of DTP3 and MCV1 in the absence of the COVID-19 pandemic was calculated on the basis of past trends in coverage and the population for 2020 (and then divided by 12 for each month). Estimated doses missed reflect the number of children who were eligible to receive a given vaccine at some point during the year (ie, the target population) but did not receive it by the end of 2020. The proportion of total doses missed attributable to the COVID-19 pandemic was then calculated by subtracting total doses missed from expected doses missed on the basis of past trends. DTP3=diphtheria-tetanus-pertussis, third dose. MCV1=measles-containing vaccine, first dose. GBD=Global Burden of Diseases, Injuries, and Risk Factors Study.

At the end of 2020, an estimated 30·0 million (95% UI 27·6–33·1) eligible children remained without doses of DTP3, as did 27·2 million (23·4–32·5) children without MCV1 doses (figure 4, table). These estimates represent an additional 8·5 million (6·5–11·6) children missing doses of DTP3 and 8·9 million (5·7–13·7) children missing doses of MCV1 in 2020 compared to expectations without the COVID-19 pandemic. Similar to coverage disruptions, the largest total number of children missing doses was in April, 2020: 4·6 million (4·0–5·4) for DTP3 and 4·4 million (3·7–5·2) for MCV1. At the end of 2020, south Asia had the highest number of additional children estimated to have missed DTP3 and MCV1 doses compared to expectations in the absence of COVID-19: 3·6 million (3·0–4·2) for DTP3 and 2·2 million (1·8–2·6) for MCV1—nearly twice as many eligible children missing doses of each vaccine as expected in the absence of the pandemic. For some GBD super-regions, including central Europe, eastern Europe, and central Asia, north Africa and the Middle East, and high-income countries, the estimated number of missed DTP3 and MCV1 doses attributable to pandemic-related disruptions more than doubled; however, less pronounced gaps associated with COVID-19 were estimated for other regions, including an estimated increase in missed doses of less than 9·6% (7·9–12·1) in sub-Saharan Africa.

## Discussion

In this study, we provide the first modelled quantitative assessment of global and regional disruptions to routine immunisation in 2020. Globally, estimated coverage in 2020 fell to 76·7% for DTP3, levels not seen since 2008, while MCV1 dropped to 78·9%, levels not seen since 2006.^8^ These COVID-19-related disruptions were most severe in the earlier months of the pandemic, reaching a nadir in April, 2020. The second half of 2020 showed signs of recovery, as global monthly doses administered began nearing expected estimates by December, 2020. Nevertheless, recovery efforts were far from complete, with an additional 8·5 million children still missing DTP3 doses and 8·9 million children still missing MCV1 doses, at the end of 2020. COVID-19 remains a formidable threat in 2021, with new variants emerging^40^ and SARS-CoV-2 transmission continuing even as countries are rapidly rolling out COVID-19 vaccines.^41^ In the absence of concerted routine immunisation catch-up and expansion efforts, especially as populations return to pre-pandemic interactions, the world will face heightened risks of vaccine-preventable diseases in 2021 and beyond.

As SARS-CoV-2 rapidly spread in March–April, 2020, disruptions to routine immunisation programmes were large and pervasive,^42^ exacerbating stalled global progress in childhood vaccination.^8^, ^43^, ^44^ In April, 2020, global routine doses of DTP3 and MCV1 fell by an estimated 30% compared to expected levels, with several GBD super-regions experiencing sharper declines. Despite disruptions to vaccination, in the short term the transmission risk of vaccine-preventable diseases might have been temporarily mitigated by mask use, physical distancing, and other types of preventive behaviours practised during the pandemic. As evidenced by past epidemics and modelling exercises,^45^, ^46^ substantive gaps in vaccine coverage increase the risk of vaccine-preventable disease outbreaks once COVID-19 prevention measures subside and individuals resume pre-pandemic social contact patterns. A recent study suggests a potential 10% increase in mortality from vaccine-preventable diseases as a result of pandemic-related disruptions to routine immunisation and other planned vaccination campaigns,^45^ while other work has associated declines in doses with increases in polio cases in endemic countries.^6^ Previous infectious disease outbreaks, such as the 2014 Ebola virus epidemic in west Africa, also led to large disruptions to routine immunisation and subsequent measles outbreaks.^47^, ^48^, ^49^ Declines in MCV1 coverage suggest growing immunity gaps and underscore the crucial need for rapid recovery of vaccination services, planned vaccination campaigns, and other catch-up activities to prevent measles outbreaks.

Vaccination services, whether at fixed sites or via outreach, might have been particularly prone to disruptions given the nature of vaccine administration—close contact between recipients and vaccinators—and the multifaceted ways in which COVID-19 affected vaccine programmes.^50^, ^51^ These compounding factors, ranging from supply and facility constraints to fear of SARS-CoV-2 exposure in health-care settings,^4^, ^5^, ^12^, ^52^ might explain why routine immunisation was among the most affected health services during earlier phases of the pandemic in 2020.^7^ The precise causes of pandemic-related disruptions to routine immunisation services—and the responses of programmes to those disruptions—have varied across and within countries. For instance, some countries sought to maintain routine immunisation services amid lockdown measures but demand nonetheless fell, while others had to temporarily reduce services as programmes faced staff re-deployment for the COVID-19 response, PPE shortages, and new safety requirements.^4^, ^51^, ^53^

As is increasingly documented in country reports^4^ and highlighted in the present analysis, recovery of vaccine delivery is underway in much of the world. Countries have adapted routine immunisation services during COVID-19 and approached catch-up efforts in ways that both leverage pre-existing programme strengths and incorporate protocols for safer vaccine administration. For instance, health workers have provided vaccine services outside clinics, including home-based visits, drive-through vaccination, and short-term vaccination posts at schools, banks, supermarkets, or pharmacies, while facilities have implemented various strategies to reduce exposure risk such as setting up tents outside health centres and extending hours or requiring appointments to reduce crowding.^51^, ^54^, ^55^, ^56^ Furthermore, as vaccination campaigns resume, activities have been altered to reduce crowding and physical contact while providing sufficient PPE.^57^, ^58^

Still, in 2021 and beyond, continued recovery of routine immunisation is far from inevitable, especially as countries face ongoing challenges in this next phase of the COVID-19 pandemic. Efforts to maintain routine immunisation services and reach children missed earlier in the pandemic will occur amid continued transmission of SARS-CoV-2 in much of the world, which is at least partly being driven by new variants; other barriers to success include the demands placed on strained health systems by mass COVID-19 vaccine roll-outs,^41^ and the exacerbation of inequalities in access to and the reach of routine immunisation against the backdrop of rising poverty in many countries.^59^

Specific strategies for recovery and expansion of routine immunisation services will vary by context, but at the global level, actions such as leveraging COVAX investments for routine immunisation functions^41^, ^60^ and broader catch-up efforts to also reach zero-dose children and underserved communities^61^, ^62^, ^63^ could start paving the way for stronger routine immunisation systems. Recovery efforts should not only accelerate and monitor catch-up vaccination initiatives but also expand services to children historically missed or underserved by routine immunisation programmes, so that pre-existing gaps in vaccine coverage do not become more entrenched.^64^ In some regions, such as sub-Saharan Africa, and many subpopulations, vaccine coverage before the pandemic was already far short of global targets and levels required to prevent disease outbreaks.^8^, ^65^ Simply recovering to pre-pandemic levels of vaccine coverage would maintain these persistent inequities and limit future gains in child health.

As underscored by the COVID-19 pandemic, having timely and granular data on where resources should be targeted is crucial for optimal response and uptake. The same applies to routine immunisation services, wherein regularly collected information on children who have missed doses can guide catch-up activities at individual and community levels.^13^, ^51^, ^66^, ^67^, ^68^ Most routine immunisation systems are not currently designed to track doses outside of their target age groups, which can limit tracking of catch-up efforts.^69^ Survey data might more fully capture both disruptions to and recovery of routine immunisation services amid COVID-19, especially as fieldwork resumes;^70^, ^71^ however, due to time lags in collection and processing of survey data, results might not be available for months or even years.^72^ Locally tailored strategies are needed now, but in the absence of timely data on past and current trends in doses and coverage, many countries could face a prolonged path to immunisation recovery. By improving routine immunisation data systems and delivery models, there could be an opportunity to build back stronger, more equitable health services for all populations.^64^ Otherwise, previous and current disruptions resulting from the COVID-19 pandemic could further deepen disparities in vaccination and child health more broadly for generations to come.

This study is subject to several limitations. First, monthly vaccine data were not available for all locations over time in 2020, and thus modelled relationships between mobility patterns and vaccine disruptions were used. Based on our validation analyses for locations with available data, modelled estimates were strongly related to observed vaccination disruptions (appendix section 4.3); nonetheless, it is possible these relationships vary for locations without data. These estimates provide an initial view of coverage disruptions in 2020, but will require iterative refinement as more data become available (eg, the annual release of Joint Reporting Form data from WHO and UNICEF^73^). Second, changes in administrative data might not correspond to changes in coverage. These data generally exclude doses provided outside of target schedules and supplemental immunisation activities, and therefore might not fully capture catch-up vaccination efforts. Additionally, we were unable to fully account for potential reporting delays or data quality challenges associated with the ongoing pandemic; furthermore, any stockouts or reporting anomalies during the baseline months could skew estimated effects of the disruption to routine immunisation. Third, mobility measures are imperfect proxies for the broader societal effects of the pandemic and might not fully represent behavioural changes in care seeking or demand for vaccination services across locations and over time. Fourth, although we incorporated uncertainty from the vaccination disruption data and from the estimated coverage in the absence of COVID-19, we did not include uncertainty in the estimates of human mobility or size of the target population for vaccination; therefore, the 95% UIs may not capture all sources of uncertainty in the analysis. Fifth, the step 1 model currently assumes constant relationships between mobility and vaccine coverage, but as illustrated by the results of the step 2 model (appendix, supplemental results section), coverage has generally increased faster than relative mobility. These patterns could reflect efforts to adapt service delivery models and conduct catch-up vaccination in ways that involve less overall population movement; examples of this approach include doing smaller outreach campaigns and home visits, and spacing clinic visits outside of typical hours. Should more data on care seeking, vaccine doses administered, and coverage by location and time become available, future analyses might be able to better capture how the relationships between service delivery and mobility have evolved during the COVID-19 crisis. Last, currently available data on vaccination in 2020 are largely limited to total aggregate numbers of doses delivered at the national level, by month. As such, our model produces aggregated estimates of disruptions to routine immunisation. The effects of the pandemic on routine immunisation coverage, however, might vary by subnational geography, sex, ethnicity or race, income, migrant or refugee status, or other important factors. In the absence of more granular data and targeted responses for recovery of routine immunisation services, COVID-19 could easily compound pre-pandemic inequities and health risks in underserved and marginalised populations. Collecting and analysing such disaggregated data, particularly for groups inadequately represented by traditional health information systems, will be important to ensure greater equity in provision of routine immunisation services.

The COVID-19 pandemic led to unparalleled disruptions in vaccine delivery, with global coverage of DTP3 and MCV1 in 2020 estimated to have fallen to levels not seen in more than a decade. Although signs of recovery emerged in the second half of 2020, the COVID-19 pandemic and its disruptive effects continue, and only returning to pre-pandemic vaccination rates would still leave millions of children under-vaccinated or unvaccinated and at risk of vaccine-preventable diseases. During the next phase of the pandemic, wherein a major focus is scaling up COVID-19 vaccines and containing new variants, routine immunisation catch-up and expansion efforts must be sustained, otherwise the world's fragile progress could easily give way to vaccine-preventable disease outbreaks in 2021 and beyond. Moving forward, the world should build upon the lessons learned about adaptive and resilient routine immunisation programmes during COVID-19 and strive to provide more equitable, sustainable vaccine services for all.

## Data sharing

Data inputs and metadata (or, for inputs that cannot be shared due to data use restrictions, relevant contact information) will be available through the Global Health Data Exchange (GHDx) upon publication at http://ghdx.healthdata.org/.

## Declaration of interests

MPV-G and RM-P are staff members of the Pan American Health Organization. The authors alone are responsible for the views expressed in this Article, and they do not necessarily represent the decisions or policies of the Pan American Health Organization. NF reports receiving funding for work unrelated to this Article from Gates Ventures since June, 2020. All other authors declare no competing interests.
