# Supplementary material for: Estimating global and regional disruptions to routine childhood vaccine coverage during the COVID-19 pandemic in 2020: a modelling study
Source: Lancet. 2021 Aug 7;398(10299):522–34. doi: 10.1016/S0140-6736(21)01337-4 (PMC8285122; doi:10.1016/S0140-6736(21)01337-4)
Supplement: Supplementary appendix [file mmc1.pdf]

THE LANCET

Supplementary appendix

This appendix formed part of the original submission and has been peer reviewed. We post it as supplied by the authors.

Supplement to: Causey K, Fullman N, Sorensen RJD, et al. Estimating global and regional disruptions to routine childhood vaccine coverage during the COVID-19 pandemic in 2020: a modelling study. *Lancet* 2021; published online July 15. http://dx.doi.org/10.1016/S0140-6736(21)01337-4.

Supplementary appendix to Estimating global and regional disruptions to routine childhood vaccine coverage during the COVID-19 pandemic in 2020: a modelling study

This appendix provides more detailed methodology and supplemental figures, tables, and results for "*Estimating global and regional disruptions to routine childhood vaccine coverage during the COVID-19 pandemic in 2020: a modelling study*." Portions of this appendix have been adapted from Galles and colleagues,[1] and as well as Zheng and colleagues.[2] References are provided for adapted sections.

Table of contents

Section 1: GATHER checklist 3
Section 2. Data processing 4
Section 2.1: Data inclusions and exclusions 4
Section 2.2: Imputation for missing vaccines 6
Section 2.3: Data variance calculations 6
Section 2.4: Subnational data 7
Section 2.5: Data coverage 8
Section 3: Spline cascade for continuous data 8
Section 3.1: Spline modeling 8
Section 3.2: Linear mixed effects model with constraints and priors 9
Section 3.3: Spline constraints 9
Section 3.4: Cascade concept and notation 10
Section 3.5: Uncertainty quantification 12
Section 4: Model details 12
Section 4.1: Modelling equations 12
Section 4.2: Uncertainty estimation 14
Section 4.3: In-sample validation 15
Section 4.4: Out-of-sample validation 16
Section 4.5: Spline parameter sensitivity analysis 20
Section 4.6: GBD vaccine coverage estimates 21
Section 4.7: Estimation of expected 2020 coverage in the absence of COVID-19 22
References 23
Supplementary results 25

Section 1: Compliance with Guidelines for Accurate and Transparent Health Estimates Reporting (GATHER)

Item #	Checklist item	Description of Compliance
**Objectives and funding**		
**1**	Define the indicator(s), populations (including age, sex, and geographic entities), and time period(s) for which estimates were made.	Introduction (main text)
**2**	List the funding sources for the work.	Funding
**Data Inputs**		
For all data inputs from multiple sources that are synthesized as part of the study:		
**3**	Describe how the data were identified and how the data were accessed.	Vaccine delivery and coverage (main text); Data inclusions and exclusions (Supplementary Appendix [SA] section 2.1)
**4**	Specify the inclusion and exclusion criteria. Identify all ad-hoc exclusions.	Vaccine delivery and coverage (main text); Data inclusions and exclusions (SA section 2.1)
**5**	Provide information on all included data sources and their main characteristics. For each data source used, report reference information or contact name/institution, population represented, data collection method, year(s) of data collection, sex and age range, diagnostic criteria or measurement method, and sample size, as relevant.	Vaccine delivery and coverage (main text); Data inclusions and exclusions (SA section 2.1); List of included data sources provided through GHDx link (upon publication); Supplementary table s1
**6**	Identify and describe any categories of input data that have potentially important biases (e.g., based on characteristics listed in item 5).	Vaccine delivery and coverage (main text); Limitations (main text); Data inclusions and exclusions (SA section 2.1)
For data inputs that contribute to the analysis but were not synthesized as part of the study:		
**7**	Describe and give sources for any other data inputs.	Human mobility (main text); GBD Vaccine Coverage Estimates and Estimation of expected 2020 coverage in the absence of COVID-19 (SA sections 4.6 and 4.7)
For all data inputs:		
**8**	Provide all data inputs in a file format from which data can be efficiently extracted (e.g., a spreadsheet rather than a PDF), including all relevant meta-data listed in item 5. For any data inputs that cannot be shared because of ethical or legal reasons, such as third-party ownership, provide a contact name or the name of the institution that retains the right to the data.	Data inputs and metadata (or, for inputs that cannot be shared due to data use restrictions, relevant contact information) will be available through GHDx upon publication
**Data analysis**		
**9**	Provide a conceptual overview of the data analysis method. A diagram may be helpful.	Methods (main text); Spline cascade for continuous data and Modelling equations (SA sections 3 and 4.1)

**10**	Provide a detailed description of all steps of the analysis, including mathematical formulae. This description should cover, as relevant, data cleaning, data pre-processing, data adjustments and weighting of data sources, and mathematical or statistical model(s).	Methods (main text); Data processing, Spline Cascade for Continuous Data, and Modelling equations (SA sections 2, 3, and 4.1)
**11**	Describe how candidate models were evaluated and how the final model(s) were selected.	Spline parameter sensitivity analysis (SA section 4.5)
**12**	Provide the results of an evaluation of model performance, if done, as well as the results of any relevant sensitivity analysis.	Estimating disruptions of routine immunisation attributable to COVID-19 (main text); In-sample validation, Out-of-sample validation, and Spline parameter sensitivity analysis (SA sections 4.3-4.5)
**13**	Describe methods for calculating uncertainty of the estimates. State which sources of uncertainty were, and were not, accounted for in the uncertainty analysis.	Estimating disruptions of routine immunisation attributable to COVID-19 and Estimating monthly number of children missing vaccine doses (main text); Uncertainty quantification, Uncertainty estimation, and GBD Vaccine Coverage Estimates (SA sections 3.5, 4.2, and 4.6)
**14**	State how analytic or statistical source code used to generate estimates can be accessed.	Available through GitHub link (upon publication)
**Results and Discussion**		
**15**	Provide published estimates in a file format from which data can be efficiently extracted.	Available through GHDx (upon publication)
**16**	Report a quantitative measure of the uncertainty of the estimates (e.g., uncertainty intervals).	Results, Figure 2, Figure 3, Table 2 (main text); Supplementary table s2
**17**	Interpret results in light of existing evidence. If updating a previous set of estimates, describe the reasons for changes in estimates.	Discussion (main text)
**18**	Discuss the limitations of the estimates. Include a discussion of any modelling assumptions or data limitations that affect interpretation of the estimates.	Limitations (main text)

Section 2: Data processing

Section 2.1: Data inclusions and exclusions

Supplementary table 1 details all sources included in the analysis, including data from the Asociación Española de Vacunología,[3] DHIS-2 in Nepal,[4] HMIS in India,[5] NCIRS in Australia,[6] NHMIS in Nigeria,[7] Public Health England,[8] Public Health Scotland,[9] Scientific American,[10] Virginia Mercury,[11] and data reported by countries to WHO Regional Offices.[12] We included data for the first dose of measles-containing-vaccine (MCV1) and third dose of diphtheria-tetanus-pertussis (DTP3) for 2020, using 2019 and/or January-February 2020 as the reference period.

Data were found through internet searches and professional contacts. We also reviewed all of the studies in a systematic review[13] and a curated list of relevant publications[14] for data that met inclusion criteria.

For the data that national governments reported to WHO regional offices, we calculated monthly cumulative disruption ratios (CDRs) based on the monthly number of doses administered in 2019-2020. For several countries in Africa (N = 22) we were missing monthly doses delivered for September-December of 2019. We imputed values for these months by subtracting the sum of all available monthly values of doses delivered (January – August) from the total annual 2019 doses delivered as reported to the WHO via the Joint Reporting Form[15] and dividing by 4 to create a monthly average estimate number of doses delivered in the last quarter of 2019.

After imputation, we calculated 1402 CDRs. We excluded CDRs that did not align with qualitative sources of disruption data,[16,17,18] likely included bias in the final month due to reporting delays, or countries for which we had an alternative source of administrative data. Exclusions are detailed in the figure below:

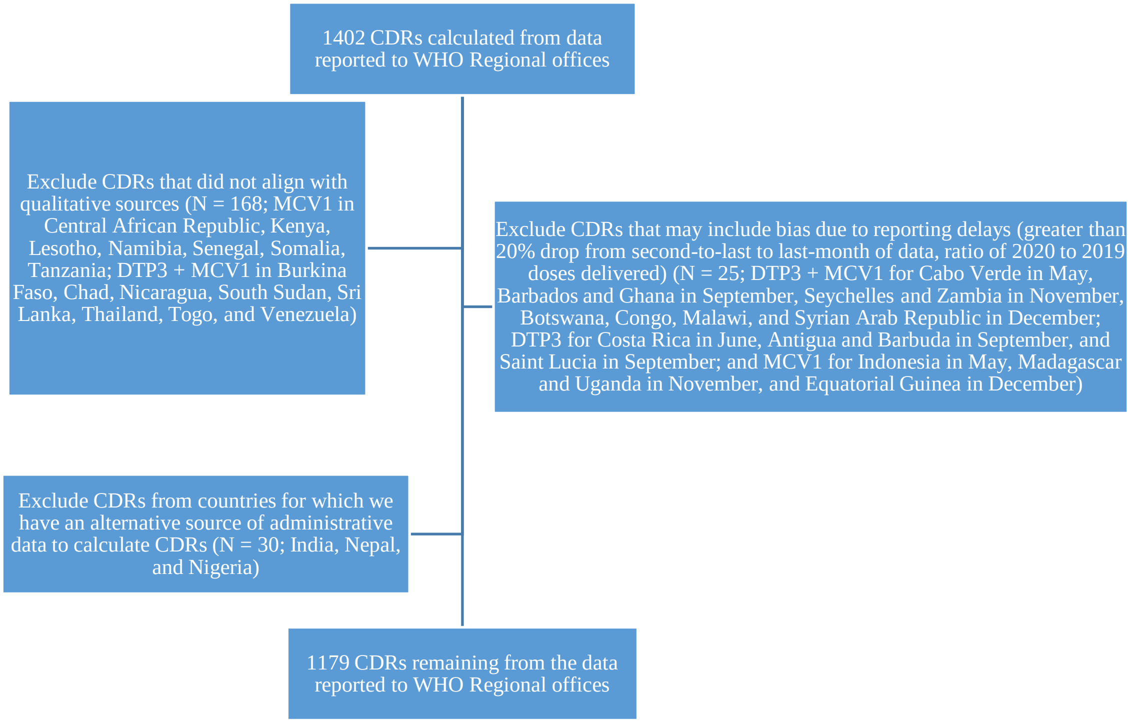

We decided to incorporate the adjustment for reporting delays after seeing evidence of lagged reporting across sequential releases of administrative data. In several countries, we saw evidence of under-reporting in the last month of available data, suggested by low numbers of doses delivered in an early data release that were adjusted upwards in a subsequent data release. As a result, we decided to exclude any CDRs from the terminal month of the time series where we saw a decrease of greater than 20% between the 2020 to 2019 ratio calculated for the second-to-last month of data compared to the ratio calculated for the last month of data.

Additionally, we conducted a sensitivity analysis, excluding all last months of data from the administrative data reported to WHO Regional offices to understand the potential impact on our results. Results from this analysis are provided in section 4.5.

The following table details the data included by Global Burden of Disease (GBD) super-region:

GBD super-region (number of countries)	Number of countries with data, N (%)	Number of CDRs									
		Mar	Apr	May	Jun	Jul	Aug	Sep	Oct	Nov	Dec
Central Europe, eastern Europe, and central Asia (29)	1 (3%)	2	2	0	0	0	0	0	0	0	0
High-income (36)	6 (17%)	46	40	10	10	10	8	8	4	4	4
Latin America and Caribbean (33)	26 (79%)	52	52	52	51	44	42	36	0	0	0
North Africa and Middle East (21)	5 (24%)	10	10	10	10	10	10	8	8	8	6
South Asia (5)	5 (100%)	10	10	10	10	4	4	4	4	4	4
Southeast Asia, east Asia, and Oceania (34)	11 (32%)	22	20	13	13	4	4	4	4	2	0
Sub-Saharan Africa (46)	40 (87%)	73	73	71	71	71	71	67	63	53	13

Section 2.2: Imputation for missing vaccines

In location-months missing one of the two vaccines, due to data exclusions or missingness, we calculated CDRs based on the available vaccine and the ratio of CDRs for DTP3 and MCV1.

For Antigua and Barbuda, Costa Rica, Equatorial Guinea, Indonesia, Madagascar, Saint Lucia, and Uganda, we had overlapping data of both vaccines to calculate location-specific ratios, MCV1 over DTP3, of 1.27, 0.57, 1.16, 1.03, 1.13, 0.92, and 1.03 respectively. To impute for Alabama (USA), Central African Republic, Kenya, Lesotho, Namibia, Senegal, Somalia, and Tanzania, we calculated a global ratio based on 608 pairs in 90 locations; the average disruption ratio was 1.01 for MCV1 to DTP3.

Section 2.3: Data variance calculations

For each data point, we assigned a weight in the model equal to the inverse variance of the log ratio.[19] For sources that provided monthly doses in 2019 and 2020 (DHIS-2 in Nepal,[4] HMIS in India,[5] NHMIS in Nigeria,[7] Public Health England,[8] Public Health Scotland,[9] and WHO Regional Offices[12]), we calculated the variance of the log cumulative ratio for month, m, with the following formula:[20]

$$Var(\log(CDR)) = \frac{1}{number\ of\ doses\ delivered\ Jan - Feb\ 2019} + \frac{1}{number\ of\ doses\ delivered\ Jan - Feb\ 2020} + \frac{1}{number\ of\ doses\ delivered\ Mar - m\ 2019} + \frac{1}{number\ of\ doses\ delivered\ Mar - m\ 2020}$$

By using this formula, the variance decreased over time as the number of doses increased. While this method provides a quantitative estimate of the variance for each administrative data point, it is difficult to fully quantify all potential sources of uncertainty in administrative data systems (i.e. due to variation in the reliability of administrative reporting systems). For instance, estimates calculated based on administrative data in large population countries had implausibly small variance due to the high number of vaccines administered. We therefore set a monthly minimum and maximum on the standard error of the log ratio, equal to the 20th and 80th percentiles of all available data by month. We used September quantities for October-December, only calculating values for months with at least 100 CDRs. This minimum affected the standard error of all 20 CDRs from the NHMIS data,[7] 256 CDRs from the WHO Regional Office data,[12] and all 8 CDRs from the India HMIS data,[5] while the maximum affected 238 CDRs from the WHO administrative data.[12]

Month	N	Median SE	20th percentile SE	80th percentile SE
**March**	177	$1.46 * 10^{-2}$	$6.58 * 10^{-3}$	$5.14 * 10^{-2}$
**April**	175	$1.23 * 10^{-2}$	$5.47 * 10^{-3}$	$4.27 * 10^{-2}$
**May**	164	$1.03 * 10^{-2}$	$4.99 * 10^{-3}$	$3.92 * 10^{-2}$
**June**	163	$0.96 * 10^{-2}$	$4.61 * 10^{-3}$	$3.73 * 10^{-2}$
**July**	141	$0.92 * 10^{-2}$	$4.55 * 10^{-3}$	$3.41 * 10^{-2}$
**August**	139	$0.89 * 10^{-2}$	$4.41 * 10^{-3}$	$3.35 * 10^{-2}$
**September**	127	$0.87 * 10^{-2}$	$4.41 * 10^{-3}$	$2.79 * 10^{-2}$

For estimates without sample size or sufficient information to calculate the variance, we imputed the median of the standard error of the log cumulative ratio by month for sources based on administrative data, electronic medical records, and immunization registries (N = 18, sources: Asociación Española de Vacunología[3] and NCIRS in Australia[6]) and the 80th percentile of the standard error of the log CDR by month for sources published in news media (N = 58, sources: Scientific American[10] and Virginia Mercury[11]). We used September quantities for October-December.

Section 2.4: Subnational data

In Spain, the United Kingdom, and the United States of America, there were not sources of nationally-representative data, but we did have access to data from some administrative regions in Spain, Scotland and England of the UK, and several US states (supplementary table 1). Because we had mobility disruption data aligned to these subnational locations, we included all subnational-level data in the step 1 national model for the corresponding country. For example, paired observations of vaccine coverage and mobility disruptions from US states were included in the step 1 model for the US to inform the relationship between vaccine CDRs and cumulative mobility disruptions (figure 2).

In the step 2 model of residuals, we only included data at the national level. In the US and the UK, the locations included represented over 50% of the national population; we population-weighted the available subnational CDRs to calculate approximate national-level CDRs for each month. The residuals were calculated at the national level as inputs to the step 2 model. For Spain, the available data did not provide sufficient coverage of the national population, so we elected to use the location-specific step 1 model and the super-region step 2 model to make estimates in Spain

Section 2.5: Data coverage

The following table enumerates the data coverage for both routine immunisation (RI) disruptions (rows) and mobility data (columns). It displays the number of countries and the fraction of the target population that fall into each data-availability category. We have mobility data in 69% of countries (*n*=134), accounting for 87% of the target population for both DTP3 and MCV1. The majority of children in the target populations for each vaccine live in countries where we have data on both changes in human mobility and RI disruptions. Less than 4% of children in the target population live in countries where we have data on neither.

**Mobility data**		**Number of countries**			**Fraction of target population DTP3**			**Fraction of target population MCV1**		
		Yes	No	**Total**	Yes	No	**Total**	Yes	No	**Total**
**RI data**	Yes	60	34	**94**	57.5%	9.3%	**66.8%**	57.3%	9.2%	**66.6%**
	No	74	36	**100**	29.4%	3.8%	**33.2%**	29.5%	3.9%	**33.4%**
	**Total**	**134**	**70**	**194**	**86.9%**	**13.1%**	**100.0%**	**86.9%**	**13.1%**	**100.0%**

Section 3: Spline cascade for continuous data

Section 3.1: Spline modeling[2]

In this section we discuss spline models for dose-response relationships. For general background on splines and spline regression see various sources.[21,22]

A spline basis is a set of piecewise polynomial functions with designated degree and domain. If we denote polynomial order by p, and the number of knots by k, we need $p + k$ basis elements s_j^p, which can be generated recursively as illustrated in the figure below.

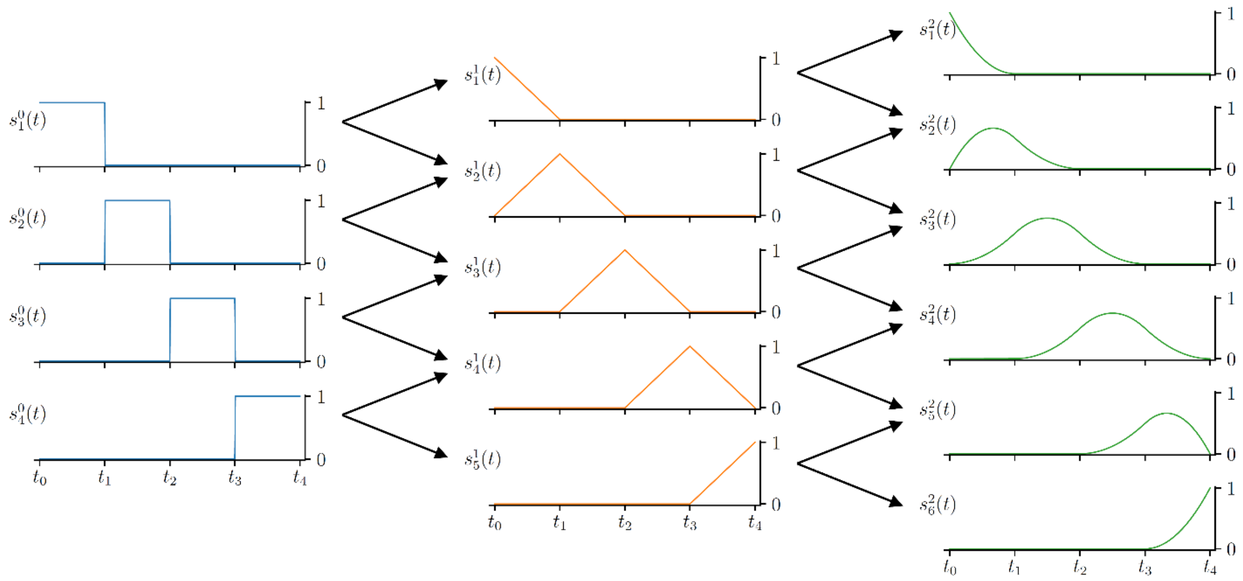

Recursive generation of bspline basis elements (orders 0, 1, 2).

Given such a basis, we can represent any dose-response relationship as the linear combination of the spline basis elements, with coefficients $\boldsymbol{\beta} \in \boldsymbol{R}^{p+k}$:

$$f(t) = \sum_{j=1}^{p+k} \beta_j^p s_j^p(t) \tag{1}$$

These coefficients are then inferred by the cascade model as discussed in section 3.4. An explicit representation of (1) is obtained by building a design matrix **X**. Given a set of t values at which we have data, the jth column of **X** is given by the expression

$$X_{\cdot,j} = \begin{bmatrix} s_j^p(t_0) \\ \vdots \\ s_j^p(t_k) \end{bmatrix} \tag{2}$$

The model for direct observations of data coming from (1) can now be written compactly as

$$y = X\beta + \epsilon$$

Section 3.2: Linear mixed effects model with constraints and priors

We consider the following linear mixed effects model:

$$\begin{gathered} y_i = X_i(\beta) + Z_i u_i + \epsilon_i \\ u_i \sim N(0, \Gamma), \qquad \Gamma = diag(\gamma), \qquad \epsilon_i \sim N(0, \Lambda) \end{gathered} \tag{3}$$

where $y_i \in R^{n_i}$ is the vector of observations from the ith study or source, $\epsilon_i \in R^{n_i}$ are measurement errors with given covariance Λ, $u_i \in R^{k_\gamma}$ are independent random effects, and $Z_i \in R^{n_i \times k_\gamma}$ is a linear map, and β are regression coefficients.

The linear mixed effects model can be extended to incorporate nonlinear inequality constraints

$$C\theta \leq c,$$

where $\theta = (\beta, \gamma)$.

We also allow priors on parameters of interest. We assume that priors are given by a functional form

$$\theta \sim \exp(-\rho(\theta))$$

The likelihood problem is then augmented and fit as described elsewhere.[2]

Section 3.3: Spline constraints

Using the constraints framework, we can impose shape constraints such as monotonicity, concavity, and convexity on splines.

Monotonicity. Spline monotonicity across the domain of interest follows from monotonicity of the spline coefficients.[22] Given coefficients β, the curve $f(t)$ in (2) is *monotonically non-decreasing* when

$$\beta_1 \leq \beta_2 \leq \cdots \leq \beta_n$$

and *monotonically non-increasing* if

$$\beta_1 \geq \beta_2 \geq \cdots \geq \beta_n$$

These are all linear inequality constraints, so easily incorporated into the framework of previously defined formulations for trimmed constrained mixed effects models.[2]

Convexity and concavity. For any twice continuously differentiable function $f : R \to R$, concavity and convexity are captured by the signs of the second derivative. Specifically, f is convex if $f''(t) > 0$ everywhere, and concave if $f''(t) \leq 0$ everywhere. We can compute $f''(t)$ for each interval, and impose linear inequality constraints on these expressions.

Rate constraints using derivatives. When fitting splines, it is useful to impose the constraint that point-wise rates of change are below plausible limits. The derivative of a spline is a linear function of the spline coefficients.[22] Therefore such a constraint is simply written as

$$X_p \beta \leq L$$

where X_p depends on the point in the domain as explained by (2), and L is the biologically plausible limit.

Enforcing linear tails. For large consumption with little data, we need the capacity to ensure that the last segment of the spline is linear, with slopes that match the adjacent segment at the knot. The estimated spline is then a best fit to the data, subject to this specification. Priors on the tails can also be provided.

Section 3.4: Cascade concept and notation

We develop notation and technical specification for a general cascade strategy useful for hierarchical models. We first set up a notation to explain the structure of the estimation.

Groups and levels. We are given a dataset consisting of tuples (y, x), levels $\ell \in \{1,2, \ldots L\}$, and groups $i = 1, \ldots, N$. For example, in the figure below, there are three levels and nine groups. In global health, level 1 may represent global data, level 2 may correspond to the super-region, and so on.

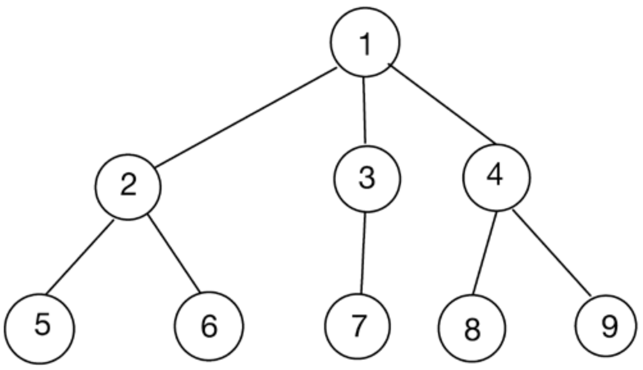

Example of a 3-level cascade with 9 groups

Every datapoint is associated to k groups, one at each level of the hierarchy. For example, in the figure above, all datapoints corresponding to group 8 at level 3 also belong to group 4 at level 2 and to group 1.

We let I_ℓ enumerate groups i at level ℓ, with $n_\ell = |I_\ell|$, and

$$N = \sum_\ell n_\ell$$

For the example in the figure above, we have $I_1 = \{1\}$, $I_2 = \{2,3,4\}$, and $I_3 = \{5,6,7,8,9\}$, so that $n_1 = 1$, $n_2 = 3$, and $n_3 = 5$.

Every group i has a unique ancestor except for the root node, whose ancestor is empty. We associate each group to its ancestor using operator ↑. For the example in the figure above, we have

$$1_\uparrow = \{\}$$

$$2_\uparrow = 3_\uparrow = 4_\uparrow = \{1\}$$

$$5_\uparrow = 6_\uparrow = \{2\}, 7_\uparrow = \{3\}, 8_\uparrow = 9_\uparrow = \{4\}$$

Modeling cascade. For every level ℓ we fit n_l models, where for each $i \in I_\ell$ we consider data associated with that group. We indicate the level ℓ using superscript notation.

$$y_i^\ell = X_i^\ell \beta_i^\ell + \epsilon_i^\ell, \qquad \epsilon_i^\ell \sim N(0, \Sigma_i^\ell) \tag{4}$$

We also allow level-specific priors on β_i^ℓ, which are informed by estimates from the parents:

$$\beta_i^\ell = \hat{\beta}_{i_\uparrow}^{\ell-1} + \nu_i^\ell, \quad \nu_i^\ell \sim N(0, \lambda^\ell V_{i_\uparrow}^{\ell-1})$$

For the special case of $\ell = 1$, we allow priors to be specified by the user. We also constrain β at all levels to lie in a set Ω, specified by equality and inequality constraints.

Translating to statements using negative log-likelihoods, for each group i we fit the model

$$\hat{\beta}_i^\ell = \arg\min_{\beta \in \Omega} g\left(\beta; y_i^\ell, \Sigma_i^\ell\right) + p(\beta; \hat{\beta}_{i_\uparrow}^{\ell-1}, \lambda^\ell V_{i_\uparrow}^{\ell-1})$$

In words, every group's fit is informed by data associated to that group along with priors whose mean and variance are obtained from those of the parent. The prior is allowed to depend on the level as well as on the parent, so that level-specific tuning parameters can be incorporated.

Gaussian example. The most common context is that of the Gaussian model (4), where we have

$$\hat{\beta}_i^\ell = \arg\min_{\beta \in \Omega} \frac{1}{2}\left(y_i^\ell - X_i^\ell \beta\right)^T \left(\Sigma_i^\ell\right)^{-1}\left(y_i^\ell - X_i^\ell \beta\right) + \frac{1}{2\lambda^\ell}\left(\beta - \hat{\beta}_{i_\uparrow}^{\ell-1}\right)^T \left(V_{i_\uparrow}^{\ell-1}\right)^{-1}\left(\beta - \hat{\beta}_{i_\uparrow}^{\ell-1}\right) \tag{5}$$

The design matrices X_i^ℓ may represent linear covariates as well as splines, which are explained in section 3.1.

Section 3.5: Uncertainty quantification

Approximate posterior uncertainty intervals can be computed for any level of the cascade using asymptotic statistics. This approach is computationally efficient but does not incorporate constraints used to define the spline.

For the key example (5), the posterior variance estimate for $\hat{\beta}_i^\ell$ is given by

$$\mathrm{Var}(\hat{\beta}_i^\ell) = \left(X_i^\ell (\Sigma_i^\ell)^{-1} X_i + \frac{1}{2\lambda^\ell}(V_{i\uparrow}^{\ell-1})^{-1}\right)^{-1}$$

The approximate asymptotic uncertainty is used to inform more detailed uncertainty quantification procedures as described in section 4.2.

Section 4: Model details

Section 4.1: Modelling equations

We modeled the relationship between human mobility and the decrease in DTP3 and MCV1 vaccine coverage with two steps of a cascading random spline model with the Meta-Regression Bayesian, Regularized, Trimmed tool (MR-BRT).[2] In step 1 we used the following set of equations to estimate the average relationship between the cumulative disruption ratio, *CDR*, and the cumulative mobility, *mob*:

**Step 1: Disruption versus Mobility**	
**Stage 1**<br>**Global model**	$\log(CDR_{m,v,c}) \sim spline(mob_{m,c}) * \boldsymbol{\beta}_1$
**Stage 2**<br>**Super-region model**	$\log(CDR_{m,v,c}) \sim spline(mob_{m,c}) * \boldsymbol{\beta}_{1,r}$<br>$\boldsymbol{\beta}_{1\,r} \sim N(\boldsymbol{\beta}_1, {\sigma_{1\,r}}^2 * \mathbf{I})$
**Stage 3**<br>**Super-region-vaccine model**	$\log(CDR_{m,v,c}) \sim spline(mob_{m,c}) * \boldsymbol{\beta}_{1\,v,r}$<br>$\boldsymbol{\beta}_{1\,v,r} \sim N(\boldsymbol{\beta}_{1\,r}, {\sigma_{1\,v,r}}^2 * \mathbf{I})$
**Stage 4**<br>**Location-vaccine model**	$\log(CDR_{m,v,c}) \sim spline(mob_{m,c}) * \boldsymbol{\beta}_{1\,v,c}$<br>$\boldsymbol{\beta}_{1\,v,c} \sim N(\boldsymbol{\beta}_{1\,v,r}, {\sigma_{1\,v,c}}^2 * \mathbf{I})$

Where *m* is the month, *v* is the vaccine – DTP3 or MCV1, *r* is the super-region, and *c* is the country. In stage 1 we fit a global model to all available ratios. We fit a cubic spline with a linear tail on the right side to the natural log of the ratio. We did not include an intercept, forcing the spline to intersect 1 (no disruption in vaccine coverage) at a mobility value of 0 (no change from baseline mobility). We also implemented a monotonicity prior, reflecting our prior assumption that vaccine coverage decreases as mobility decreases.

Each stage fit the same model to a subset of data, leveraging the estimates from the previous stage as a prior on the subsequent stage. In this way, the global model informed the super-region models; the super-region model informed the super-region-vaccine-specific model; and the super-region-vaccine-specific model informed the location-vaccine-specific model.

In the equations above, items in bold ($\boldsymbol{\beta}$) represent matrices, and **I** is the identity matrix. Other values including (CDR, mob, and σ) are vectors, and $N()$ represents the Gaussian distribution. The notation $spline()$ refers to the design matrix described in section 3.2 equation 2.

While the step 1 model estimates the average relationship throughout the pandemic, it fails to account for the dynamic country-response over time. In step 2, we estimated the deviation from the average relationship over time by calculating the residuals between the data and model predictions over time. We used the following set of equations to estimate the residual, *r*, by month, *m*:

**Step 2: Residuals over time**	
**Calculate Residuals**	$r_{m,v,c} = logit(CDR_{m,v,c}) - logit(p_{m,v,c})$
**Stage 1**<br>**Global model**	$r_{m,v,c} \sim spline(m) * \boldsymbol{\beta}_2 + \beta_0 + u_c$<br>$u_c \sim N(0, \sigma_{3\,g}^2)$
**Stage 2**<br>**Super-region model**	$r_{m,v,c} \sim spline(m) * \boldsymbol{\beta}_{2\,r} + \beta_{0\,r} + u_{c\,r}$<br>$\boldsymbol{\beta}_{2\,r} \sim N(\boldsymbol{\beta}_2, {\sigma_{2\,r}}^2 * \mathbf{I})$<br>$u_{c\,r} \sim N(0, \sigma_{3\,r}^2)$
**Stage 3**<br>**Super-region-vaccine model**	$r_{m,v,c} \sim spline(m) * \boldsymbol{\beta}_{2\,v,r} + \beta_{0\,v,r} + u_{c\,v,r}$<br>$\boldsymbol{\beta}_{2\,a,r} \sim N(\boldsymbol{\beta}_{2\,r}, {\sigma_{2\,v,r}}^2 * \mathbf{I})$<br>$u_{c\,v,r} \sim N(0, \sigma_{3\,v,r}^2)$
**Stage 4**<br>**Location-vaccine model**	$r_{m,v,c} \sim spline(m) * \boldsymbol{\beta}_{2\,v,c} + \beta_{0\,v,c}$<br>$\boldsymbol{\beta}_{2\,v,c} \sim N(\boldsymbol{\beta}_{2\,v,r}, {\sigma_{2\,v,c}}^2 * \mathbf{I})$

Where *m* is the month, *r* is the residual in logit space, and *p* is the prediction from the step 1 stage 4 results. Again fitting separate models by vaccine, we fit a global model to all available residuals, using a quadratic spline with linear tails. We included an intercept, β_0, in all three stages and a location random effect, u_c, in stages with multiple locations (1-3).

When calculating the residuals we capped the residuals at the 90th percentile of the observed values (and set a minimum at the negative of this value) to reduce the influence of extreme observations. We calculated the standard error by a series of two delta transformations, converting the standard error of the log CDR first to linear space and secondly to logit space. Due to extreme values in these standard errors, we set bounds at the 25th and 75th percentiles of the standard errors.

For both steps we selected knots by taking evenly spaced quantiles of the dependent variable at the global level. For step 1 the knots fell at cumulative mobility disruptions of 0.16, 0.23, 0.30, and 0.40, and for step 2 the knots fell at 4, 6, and 8 (ie, April, June, and August).

We manually selected several other parameters to inform the spline models. Theta is a parameter that informs the strength of the prior when moving from one stage to the next in each step of the cascade. For step 1 we selected thetas of 4, 1, and 100 when moving between stages 1 and 2, stages 2 and 3, and stages 3 and 4, respectively. For step 2, we selected thetas of 20, 1, and 100. A smaller theta leads to a larger emphasis on the $\boldsymbol{\beta}$ from the earlier stage of the model in the estimation of $\boldsymbol{\beta}$ for the next stage. We also placed a prior on the maximum derivative of each spline segment with a Gaussian distribution with a mean of zero and standard error of one for step 1, stages 1-4. In step 2 we trimmed 10% of the data in the stage 1 model. In stages 2-4, we set a prior on the maximum derivative of each spline segment with a Gaussian distribution with a mean of zero and a standard error of 0.2. For the slope of the right linear tail in step 2 stages 2-4, we set a prior based on the slope value estimated in the step 2 stage 1 model: a

Gaussian distribution with a mean of -0.013 and a standard error of 0.2. This prior prevented extreme trends in the residuals in locations without data to support such trends.

We ran sensitivity analyses to examine the choice of thetas and strength of the Gaussian derivative prior. These results are provided in section 4.5.

In 94 countries with available data, we fit each step using the β estimates from each stage as a prior for the subsequent step, such that the estimates for the super-region drew strength from the global model, and the country-specific estimates drew strength from the regional and global models. The country-specific splines, therefore, only varied from the shape and magnitude of the global model if there was sufficient evidence to suggest a deviation.

In order to account for catch-up vaccination, we estimated all models in cumulative space from March 1, 2020. We chose March 1 because nearly every country experienced effects of COVID-19 on mobility by March. By using a *log* transformation and a monotonicity constraint in the step 1 model and a *logit* transformation in the step 2 model, we constrained the cumulative disruption between 0 and 1; therefore, in any one month, a country can exceed the expected rate of vaccine delivery (e.g. successfully implementing catch-up vaccination), but over time the estimated total vaccine delivery cannot exceed that expected in the absence of COVID-19.

Section 4.2: Uncertainty estimation

To calculate uncertainty intervals of disruption rates, we generated 1000 step 1 splines for each location based on the asymptotic posterior uncertainty intervals described in section 3.5 and a lognormal distribution. For countries with data, we generated a posterior set of 1000 splines based on asymptotic sampling from the mean and covariance matrix of the stage 3 splines. For countries without data, we randomly sampled with replacement from all 94 country-specific models to generate 1000 iterations. We included a sampling weight such that countries within the same super-region were 10 times as likely to be sampled as countries outside of the super-region.

For step 2, for 93 locations with nationally representative data, we similarly generated 1000 splines based on the stage 4 model, relying on asymptotic posterior uncertainty intervals. For countries without data, we generated 1000 splines from the super-region and vaccine-specific step 2 stage 3 model. Due to data sparsity (N = 4) in central Europe, eastern Europe, and central Asia, for locations without data in this region, we relied on the step 2 stage 1 global model to estimate the residuals.

Based on the sum of these two sets of 1000 draws in logit space, we generated estimates based on the mean and provided 95% uncertainty intervals.

Section 4.3: In-sample validation

For each country, vaccine, and data source combination we calculated the average vaccine disruption ratio over the full time-period available to compare to the model results for the same location, vaccine, and time-period, by step. We calculated the weighted mean error (wME), weighted mean absolute error (wMAE), and root-mean-square error (RMSE) for each vaccine and step to evaluate the bias and goodness of fits. Step 1 involved modelling the relationship between cumulative disruptions in mobility and vaccine coverage, and step 2 incorporated residual variation. The size of each point is proportional to the inverse variance of the log ratio, the same weights used in fitting the model and calculating the wME and wMAE. The results are displayed in the figure and table below:

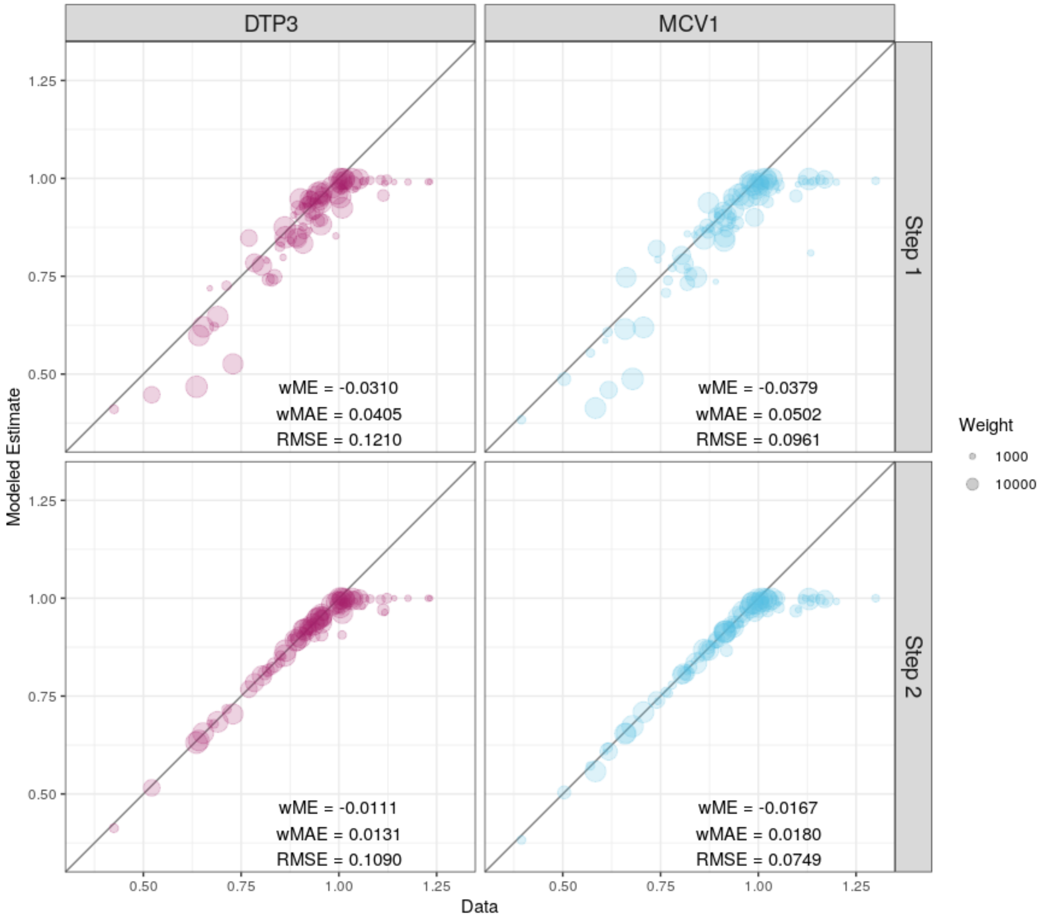

	DTP3			MCV1		
	**wME**	**wMAE**	**RMSE**	**wME**	**wMAE**	**RMSE**
**Step 1**	-3.10%	4.05%	12.10%	-3.79%	5.02%	9.61%
**Final Model**	-1.11%	1.31%	10.90%	-1.67%	1.80%	7.49%

Due to the design of our model, predictions are constrained between zero and one. This aligns with our prior understanding that we do not believe any country would be likely to exceed expected coverage in the absence of COVID-19 under the conditions and challenges that the COVID-19 pandemic presented in 2020. We performed the same in-sample validation but held out all cumulative ratios over 1 and found that much of the bias (negative sign in the weighted mean error) is accounted for by CDRs over 1. The results are enumerated in the table below, only including CDRs < 1:

	**DTP3**			**MCV1**		
	**wME**	**wMAE**	**RMSE**	**wME**	**wMAE**	**RMSE**
**Step 1**	-2.76%	4.06%	5.51%	-3.01%	4.65%	5.89%
**Final Model**	-0.27%	0.55%	1.17%	-0.36%	0.55%	1.03%

Section 4.4: Out-of-sample validation

We conducted two out-of-sample validation analyses to understand A) model performance when projecting to future months in locations with a partial time-series and B) model performance in locations without data. We used a stratified random sampling procedure to generate 20% holdouts, stratified by super-region.

In the first analysis, we aimed to estimate model performance when projecting in locations where we do not have a full time series (March-December) of data. First, as described above, we created a cross validation environment by generating 5 randomly selected holdout groups of countries, stratified by super-region. In each holdout group, we first withheld the last month of data from each country and ran a model. We then progressively held additional months of data, re-running models to produce out-of-sample estimates for each country. This produced 9 sets of out-of-sample validation models, withholding the terminal 1, 2, 3, …, and up to 9 months of data, respectively. For each country, we then compared the CDR from the terminal month in the full dataset to the out-of-sample predicted CDR for the same month after withholding *n* months. In this comparison, we only included countries for which we had at least 1 month of national-level data included in the model after holdouts. For example, 90 countries had national-level data for at least two months (March and April), so when dropping *n*=1 month of data, 90 countries were included in the comparison. Meanwhile, 43 countries had national-level data for at least 8 months (through at least October), so when dropping *n*=7 months of data, 43 countries were included in the comparison.

In the following plots, we show the number of countries (N), weighted mean error (wME), weighted mean absolute error (wMAE), and the root mean square error (RMSE) for each vaccine across the number of months withheld. We used the inverse variance of the log ratio as weights to calculate wMAE and wME. As expected, the wMAE are lowest when only withholding one month of data ($n = 1$), but the results remain fairly consistent as *n* increases, even when we withhold the terminal 7+ months of data. This analysis suggests that our model performs well when projecting forward into time for countries where we only have a partial time-series of data included in the analysis.

As previously discussed in section 4.3, the design of the model constrains CDRs between zero and one. As in section 4.3, we performed a sensitivity analysis to evaluate the influence of this constraint on model fit, calculating out-of-sample wMAE, wME, and RMSE only for countries with CDRs < 1. This sensitivity analysis resulted in wME values closer to zero, suggesting that the negative bias is at least in part attributable to the presence of CDRs < 1 in the original dataset.

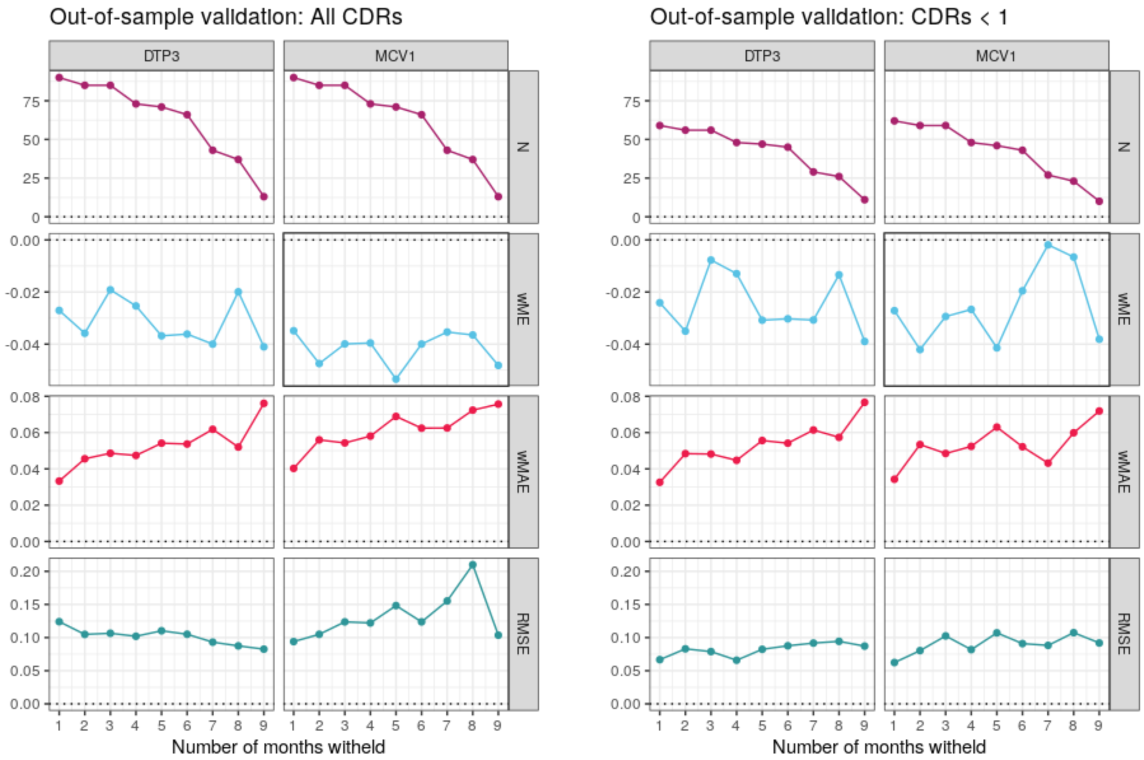

In the second analysis, we relied on the same stratified random sample of 20% holdouts but held out the entire time-series for each country in the group. This analysis serves to evaluate the performance of the model in locations where we have no data. Predictably, the model performance decreases in the absence of any data. This model is depicted in the figure below:

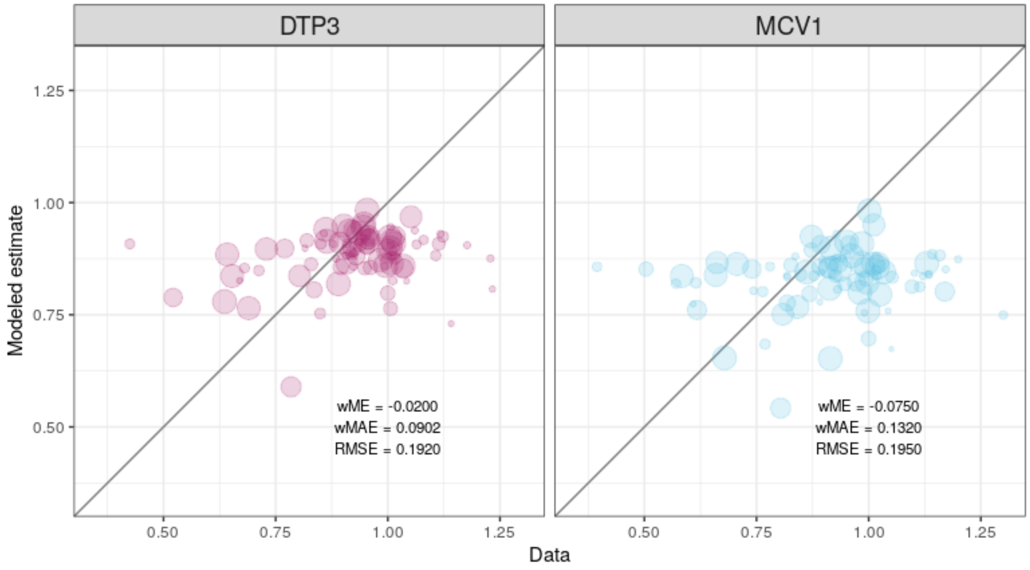

As the plot above illustrates, the out-of-sample model predictions tend to fall in a narrower range than the withheld data. Given the hierarchical nature of our model, this is to be expected: when no vaccination disruption data is available for a given country, the model uses the super-regional relationships between mobility and coverage disruption (from step 1 and step 2 of the model), along with country-specific mobility data, to predict disruptions. Within each out-of-sample model, all withheld countries for each super-region will therefore use these same super-regional relationships, with additional variation coming only from the withheld countries' mobility patterns. This process borrows strength within super-regions to produce predictions in countries without data, but as shown here may underrepresent true country-level variability in disruption. When aggregating to global or super-regional estimates, however, countries with under-estimated disruptions may be offset by countries with over-estimated disruptions during the aggregation process, as long as the model is generally unbiased.

We again repeated the same full-country-exclusion out-of-sample validation analysis excluding all CDRs over 1, as described for the partial-time-series out-of-sample validation analysis above and the in-sample validation analysis in section 4.3. Again, we find that much of the negative bias in this out-of-sample validation exercise can be attributed to the presence of CDRs > 1 in the original dataset. The results of this analysis are provided in the table below:

	**DTP3**			**MCV1**		
	**wME**	**wMAE**	**RMSE**	**wME**	**wMAE**	**RMSE**
**All CDRs**<br>n = 91, DTP3<br>n = 91, MCV1	-2.00%	9.02%	19.20%	-7.50%	13.2%	19.50%
**CDRs <1**<br>n = 59, DTP3<br>n = 62, MCV1	2.09%	7.56%	11.10%	-3.81%	11.4%	14.00%

The majority of children eligible to receive doses of DTP3 and MCV1 live in countries with at least a partial time series of data (the scenario evaluated in the first of these out-of-sample validation analyses). Globally, two out of every three children in the target populations for each DTP3 and MCV1 live in one of the 94 countries that have data included in this analysis. The following table details the fraction of the target population for each antigen who live in countries with data included in the analysis, globally and by GBD super-region:

Location	Fraction of the target population living in a country with data included in the analysis	
	**DTP3**	**MCV1**
**Global**	**66.8%**	**66.6%**
Central Europe, eastern Europe, and central Asia	1.6%	1.5%
High-income	49.6%	49.3%
Latin America and Caribbean	37.8%	37.6%
North Africa and Middle East	39.0%	38.4%
South Asia	100.0%	100.0%
Southeast Asia, east Asia, and Oceania	34.1%	34.1%
Sub-Saharan Africa	93.0%	93.0%

Section 4.5: Spline parameter sensitivity analysis

We ran the following sensitivity analyses to understand the impact of the spline parameters on the results:

Analysis description	DTP3			MCV1		
	**Global annual relative disruption (95% Uncertainty Interval)**	**wME**	**wMAE**	**Global annual relative disruption (95% Uncertainty Interval)**	**wME**	**wMAE**
**Manuscript analysis**<br>**Thetas:**<br>Step 1: 4, 1, 100<br>Step 2: 20, 1, 100<br>**Maximum derivative SE:**<br>Step 1: 1<br>Step 2: 0.2	**7.7% (6.0—10.1)**	**-1.11%**	**1.31%**	**7.9% (5.2—11.7)**	**-1.67%**	**1.80%**
**Greater influence of global and regional models**<br>**Thetas:**<br>Step 1: 2, 0.5, 50<br>Step 2: 10, 0.5, 50	7.1% (5.7—9.0)	-1.26%	1.49%	8.4% (6.0—12.3)	-2.06%	2.17%
**Weaker influence of global and regional models**<br>**Thetas:**<br>Step 1: 8, 2, 200<br>Step 2: 40, 2, 200	8.2% (6.0—11.4)	-1.07%	1.28%	8.3% (5.5—12.3)	-1.61%	1.76%
**More flexible**<br>**Maximum derivative SE:**<br>Step 1: 2<br>Step 2: 0.4	7.5% (5.2—10.7)	-0.92%	1.18%	7.9% (6.2—10.6)	-1.55%	1.73%
**Less flexible**<br>**Maximum derivative SE:**<br>Step 1: 0.5<br>Step 2: 0.1	8.7% (7.0—11.4)	-1.49%	1.68%	8.7% (6.1—13.3)	-1.99%	2.13%
**Examining bias correction**<br>Dropping all last months of admin data reported to WHO	8.8% (6.9—11.5)	-1.01%	1.25%	9.3% (6.5—13.8)	-1.65%	1.87%

As shown in this table, the global results were only somewhat sensitive to these model parameters. Though some settings (weaker influence of global and regional models and more flexible splines) improved in-sample validation criteria (wME and wMAE), increased model flexibility raises the risk of

overfitting available data. The values selected in the manuscript analysis balanced our prior expectations and global trends with location-specific results to ensure outliers did not overly influence the model.

The final row of the table presents a sensitivity analysis conducted to examine the potential effects of bias due to reporting delays in the last month of administrative data reported to the WHO regional offices. In the primary analysis, we attempt to limit the effects of this bias by excluding particular countries with evidence of large decreases (greater than 20%) in the last month of available data (section 2.1). To understand the impact of this strategy and to understand the potential impact of reporting delays, we re-ran the analysis dropping all data from the last month reported to the WHO regional offices. The results were fairly robust to this change with an increase in the global annual disruption of about 1% for both antigens; uncertainty intervals for the primary (manuscript) analysis and sensitivity analysis broadly overlapped. Eliminating the last month of data for all countries in this sensitivity analysis systematically excludes data points that suggest ongoing catch-up vaccination and/or recovery of services in the latter months of 2020. This sensitivity analysis therefore results in a larger disruption estimate (and potential over-estimation of the true magnitude of disruption) compared to the primary analysis presented in the manuscript.

Section 4.6: GBD vaccine coverage estimates[1]

Annual estimates of childhood routine vaccination coverage, including DTP3 and MCV1 are produced as part of the larger Global Burden of Diseases, Injuries, and Risk Factors Study (GBD).[23–25] The methodology for producing these estimates has been described elsewhere in detail and is adapted below.[1]

Vaccination coverage was defined as "the proportion of children that received at least the stated number of doses (e.g. DTP1) from a routine immunisation delivery system."[1] The GBD estimates leveraged survey microdata from children ages 12 months through 59 months, survey report data in the absence of microdata, and bias-adjusted official country-reported estimates available from the 2020 Joint Reporting Form (JRF).[15] We excluded doses delivered through vaccination campaigns whenever possible. Data was assigned to year of expected delivery using national immunisation schedules from the JRF,[26] assuming negligible effects of any catch-up vaccination, migration, or differential mortality in older age cohorts. In addition to direct coverage data, data on country-reported stockouts or disruptions to routine delivery were also incorporated.[15] These data were used to compute a covariate used in modelling which captured the expected magnitude of delivery disruption proportional to the change in bias-adjusted official country-reported data in years when a reported stockout was matched by a decrease in reported coverage.

Spatio-temporal Gaussian process regression (ST-GPR) was the primary modelling tool used for this analysis. Details on ST-GPR estimation for the GBD has been described elsewhere.[24] In brief, ST-GPR is a stochastic modelling framework designed to incorporate diverse input data and predict over space and time. It uses a selected linear regression as the basis of every model, followed by locally-weighted regression (LOESS) to smooth and minimise prediction error, then Gaussian process regression (GPR) to predict smoothed estimates over space and time while accounting for underlying data uncertainty. DTP3 and MCV1 were informed by the following linear model:

$$logit(P_c(t)) = \beta_0 + \beta_1 HAQ_{c,t} + \beta_2 war_{c,t} + \beta_3 stockout_{c,t} + \alpha_c + \gamma_{R[c]} + \omega_{SR[c]} + \epsilon_{c,t}$$

where $P_c(t)$ is vaccination coverage for country c year t; $HAQ_{c,t}$ is value of the health care access and quality (HAQ) index[27] for country c and year t; $war_{c,t}$ is log-transformed mortality rate due to acute war and terror events; $stockout_{c,t}$ is value of the vaccine stockout covariate for country c and year t; and α_c, $\gamma_{R[c]}$, and $\omega_{SR[c]}$ are nested country, region, and super-region random intercepts, respectively.

From the DTP3 and MCV1 models, respectively, we obtained 1,000 samples (draws) from the posterior distribution of coverage. Draws were summarised to produce mean estimates of annual vaccination coverage complemented by 95% uncertainty intervals based on the 2.5th and 97.5th ordinal draws. Estimates were produced globally for 204 countries and territories, 1980 to 2019.

Section 4.7: Estimation of expected 2020 coverage in the absence of COVID-19

Using the ST-GPR estimation framework described in section 4.6 above, we assumed that in the absence of COVID-19, country-specific national coverage trends estimated from 1980-2019 were expected to continue in the absence of the pandemic. In order to produce these estimates, we fit ST-GPR models to available vaccine coverage data from 1980-2019 as described above. We then used the fitted models to produce predictions for 2020, using modeled estimates of the HAQ index in 2020 – representing expected values of HAQ in the absence of the COVID pandemic – as a covariate. This process produced a set of estimates of vaccine coverage in the absence of COVID-19 for 2020, assuming that past trends in coverage (and HAQI) would have continued into the future. In the 2020 counterfactual, we did not include effects of any national-level vaccine stockouts nor disruptions due to war and terror given the absence of available data.

References

1 Galles NC, GBD 2020 Vaccine Coverage Collaborators. Measuring routine childhood vaccination coverage in 204 countries and territories, 1980-2019: a systematic analysis for the Global Burden of Disease Study 2020, Release 1. *The Lancet* 2021; published online Feb.

2 Zheng P, Barber R, Sorensen R, Murray C, Aravkin A. Trimmed Constrained Mixed Effects Models: Formulations and Algorithms. *J Comput Graph Stud* 2021; **0**: 1–13.

3 Moreno Pérez D. Vacunas en todas las edades: Que el COVID-19 no nos frene. 2020; published online June 2. https://www.vacunas.org/webinar-vacunas-en-todas-las-edades-que-el-covid-19-no-nos-frene/ (accessed Feb 5, 2021).

4 Department of Health Services, Ministry of Health and Population. Nepal Monthly Immunization Coverage by Province 2019-2020. Nepal: Department of Health Services, Ministry of Health and Population.

5 Ministry of Health and Family Welfare. India HMIS Standard Report - Data Itemwise Monthly 2018-2021. New Delhi, India: Ministry of Health and Family Welfare https://nrhm-mis.nic.in/hmisreports/frmstandard_reports.aspx.

6 National Centre for Immunization Research and Surveillance. COVID-19: Impact on routine childhood vaccination uptake in Australia. Sydney, Australia: National Centre for Immunization Research and Surveillance, 2020 https://ncirs.org.au/sites/default/files/2020-11/COVID-19_Impact_Analysis_Final%20Report.pdf (accessed Nov 30, 2020).

7 National Health Analytical Tool showing Health Service Uptake during the COVID-19 pandemic. Nigerian National Health Management Information System https://public.tableau.com/views/COVID19AnalysisofHealthServiceUptakeusingNHMISData/MAINDashboard (accessed March 1, 2021).

8 Public Health England. Impact of COVID-19 on childhood vaccination counts up to week 11, 2021. London, England: Public Health England, 2021 https://www.gov.uk/government/publications/covid-19-impact-on-vaccination-programmes/impact-of-covid-19-on-childhood-vaccination-counts-up-to-week-11-2021.

9 COVID-19 Wider Impacts Tool. Public Health Scotland https://scotland.shinyapps.io/phs-covid-wider-impact/ (accessed March 1, 2021).

10 Daley J. Vaccinations Have Sharply Declined Nationwide during the COVID-19 Pandemic. Sci. Am. https://www.scientificamerican.com/article/vaccinations-have-sharply-declined-nationwide-during-the-covid-19-pandemic/ (accessed Aug 13, 2020).

11 Masters K, April 27 VM, 2020. Virginia pediatricians say reported decline in vaccination rates amid COVID-19 pandemic is troubling. Va. Mercury. 2020; published online April 27. https://www.virginiamercury.com/2020/04/27/virginia-pediatricians-report-troubling-decline-in-vaccination-rates-as-covid-19-pandemic-continues/ (accessed Nov 30, 2020).

12 UNICEF, World Health Organization. Progress and Challenges with Achieving Universal Immunization Coverage: 2019 WHO/UNICEF Estimates of National Immunization Coverage [Unpublished Data]. UNICEF/WHO, 2020 https://www.who.int/immunization/monitoring_surveillance/who-immuniz.pdf.

13 Lassi ZS, Naseem R, Salam RA, Siddiqui F, Das JK. The Impact of the COVID-19 Pandemic on Immunization Campaigns and Programs: A Systematic Review. *Int J Environ Res Public Health* 2021; **18**. DOI:10.3390/ijerph18030988.

14 Danovaro MC. The impact of COVID-19 on immunization programmes - publications. TechNet-21. https://www.technet-21.org/en/topics/covid-19-impact (accessed May 11, 2021).

15 WHO/UNICEF Joint Reporting Process. WHO http://www.who.int/immunization/monitoring_surveillance/routine/reporting/en/ (accessed April 8, 2021).

16 World Health Organization. Special feature: Immunization and COVID-19: Second pulse poll to help understand disruptions to vaccination and how to respond. Geneva, Switzerland: WHO Global Immunization News (GIN) Newsletter, 2020.

17 World Health Organization. Pulse survey on continuity of essential health services during the COVID-19 pandemic. Geneva, Switzerland: World Health Organization, 2020.

18 Danovaro C, Gurung S, Ho LL, Linstrand A. Understanding the disruption to programmes through rapid polling. WHO Global Immunization News (GIN) Newsletter, 2020.

19 Lee CH, Cook S, Lee JS, Han B. Comparison of Two Meta-Analysis Methods: Inverse-Variance-Weighted Average and Weighted Sum of Z-Scores. *Genomics Inform* 2016; **14**: 173–80.

20 Bland JM, Altman DG. The odds ratio. *BMJ* 2000; **320**: 1468.

21 Friedman JH. Multivariate Adaptive Regression Splines. *Ann Stat* 1991; **19**: 1–67.

22 Boor C de, DeBoor C. A Practical Guide to Splines. Springer New York, 1978.

23 Vos T, Lim SS, Abbafati C, *et al.* Global burden of 369 diseases and injuries in 204 countries and territories, 1990–2019: a systematic analysis for the Global Burden of Disease Study 2019. *The Lancet* 2020; **396**: 1204–22.

24 Murray CJL, Aravkin AY, Zheng P, *et al.* Global burden of 87 risk factors in 204 countries and territories, 1990–2019: a systematic analysis for the Global Burden of Disease Study 2019. *The Lancet* 2020; **396**: 1223–49.

25 Wang H, Abbas KM, Abbasifard M, *et al.* Global age-sex-specific fertility, mortality, healthy life expectancy (HALE), and population estimates in 204 countries and territories, 1950–2019: a comprehensive demographic analysis for the Global Burden of Disease Study 2019. *The Lancet* 2020; **396**: 1160–203.

26 World Health Organization. WHO and UNICEF Reported Immunization Schedules 2000-2019. Geneva, Switzerland: World Health Organization, 2020.

27 Fullman N, Yearwood J, Abay SM, *et al.* Measuring performance on the Healthcare Access and Quality Index for 195 countries and territories and selected subnational locations: a systematic analysis from the Global Burden of Disease Study 2016. *The Lancet* 2018; **391**: 2236–71.

Supplementary results

Comparing vaccine and mobility patterns

The global step 1 model of cumulative vaccine disruption versus cumulative mobility disruption suggested steep declines in vaccine coverage at initial mobility decreases and continued declines through the entire range of cumulative mobility disruption. Data suggested reductions in vaccine coverage even at low levels of mobility reduction. In step 1, we found a wide range of vaccine disruption ratios across countries with similar mobility disruptions. At a 40% cumulative disruption in mobility, DTP3 vaccine disruption estimates across locations ranged from 20.0% of expected to no disruption, with a standard deviation of 16.6%. For MCV1, disruption estimates ranged from 17.7% of expected to no disruption, with a standard deviation of 18.1%.

The global step 2 model of residuals over time, suggest that recovery in vaccine delivery is outpacing that which would be expected from the relationship with mobility alone. Though the model does not distinguish doses delivered to children missed early in the pandemic from those delivered on schedule as services resume, in later months, positive residuals show that disruptions tend to be smaller compared the disruptions predicted by mobility trends. This suggests that active efforts to mitigate RI disruptions may be helping to accelerate the pace of recovery in many locations.

Supplementary tables

Supplementary table 1. Data sources for vaccine delivery disruption and metadata. AL=Alabama. AR=Arkansas. CA=California. CDR=Cumulative Disruption Ratio. CO=Colorado. DHIS-2=District Health Information Software 2. DTP=diphtheria, tetanus, and pertussis. DTP3=diphtheria, tetanus, and pertussis, third dose. FL=Florida. HMIS=Health Management Information System. IA=Iowa. ID=Idaho. IL=Illinois. KS=Kansas. KY=Kentucky. MA=Massachusetts. MCV1=measles-containing vaccine, first dose. MI=Michigan. MMR=measles, mumps, and rubella. MMR1=measles, mumps, and rubella, first dose. MN=Minnesota. MR=measles and rubella. NCIRS=National Centre for Immunisation Research and Surveillance. ND=North Dakota. NE=Nebraska. NHMIS=National Health Management Information System. NJ=New Jersey. NV=Nevada. OK=Oklahoma. OR=Oregon. PCV3=pneumococcal conjugate vaccine, third dose. Penta3=pentavalent vaccine, third dose; covers diphtheria, tetanus, pertussis, hepatitis B, and *Haemophilus influenzae* type b. RI=Rhode Island. SC=South Carolina. SD=South Dakota. TN=Tennessee. TX=Texas. USA=United States of America. UT=Utah. VA=Virginia. VT=Vermont. WI=Wisconsin.

Supplementary table 2. Expected DTP3 and MCV1 coverage in the absence of COVID-19 and estimates of disruptions and coverage attributable to the pandemic in 2020, by country. Expected coverage was expected levels for 2020 in the absence of the COVID-19 pandemic based on past trends. Estimated coverage reflects coverage for 2020 while accounting for estimated pandemic-related disruptions; more detail on these methods is in the main manuscript and appendix section 4. DTP3=diphtheria, tetanus, and pertussis, third dose. MCV1=measles-containing vaccine, first dose. UI=uncertainty interval.

Supplementary table 1. Data sources for vaccine delivery disruption and metadata.

Source	Number of CDRs	Sample size	Antigen(s)	Age (months)	Location(s)	Data source	Reference period	Reference value	Pandemic period	Pandemic value
Asociación Española de Vacunología	2	–	Hexavalent<br>MMR	0 – 15	Community of Madrid, Spain	Administrative data	Jan 2019 – Feb 2020	Monthly average number of doses administered	Mar 2020	Monthly number of doses administered
	4			–	Murcia, Spain		Monthly; Mar – Apr 2019	Monthly vaccine coverage	Monthly; Mar - Apr 2020	Monthly vaccine coverage
	2			0 – 11<br>0 – 12	Andalucía, Spain		Mar 2019	Monthly vaccine coverage	Mar 2020	Monthly vaccine coverage
DHIS-2	20	2,040,000 doses administered	DTP3<br>MCV1	–	Nepal	Administrative data	Jan 2019 – Feb 2020	Ratio of doses administered in Jan – Feb 2020 and doses administered in Jan – Feb 2019	Monthly; Mar – Dec 2020	Ratio of monthly doses administered and doses administered in the same month of 2019
HMIS	8	48,400,000 doses administered	DTP3 + Penta3<br>Measles + MR + MMR	–	India	Administrative data	Jan 2019 – Feb 2020	Ratio of doses administered in Jan – Feb 2020 and doses administered in Jan – Feb 2019	Monthly; Mar – Jun 2020	Ratio of monthly doses administered and doses administered in the same month of 2019
NCIRS	10	–	MMR<br>PCV3	0 – 14	Australia	Australia Immunization Register	Jan – Feb 2020	Vaccination uptake (%)	Monthly; Mar – Jul 2020	Vaccination uptake (%)
NHMIS	20	26,000,000 doses administered	MCV1<br>Penta3	–	Nigeria	Administrative data	Jan 2019 – Feb 2020	Ratio of doses administered in Jan – Feb 2020 and doses administered in Jan – Feb 2019	Monthly; Mar – Dec 2020	Ratio of monthly doses administered and doses administered in the same month of 2019
Public Health England	20	818,000 doses administered	Hexavalent<br>MMR	0 – 6<br>12 – 18	England	Electronic patient records	Jan 2019 – Feb 2020	Ratio of doses administered in Jan – Feb 2020 and doses administered in Jan – Feb 2019	Monthly; Mar – Dec, 2020	Ratio of monthly doses administered and doses administered in the same month of 2019
Public Health Scotland	20	74,600 doses administered	6-in-1 third dose<br>MMR1	0 – 20<br>0 – 5	Scotland	Scottish Immunisation and Recall System	Jan – Feb 2020	Average monthly number of children who had received the vaccine	Monthly; Mar – Dec, 2020	Number of children who had received their vaccine
Scientific American	56	–	Different antigens reported for each state including all, all non-influenza, MMR, and combinations of 9, 7, or 11 childhood vaccines	0 – 18<br>0 – 23<br>0 – 35<br>(state-specific)	27 states (AL, AR, CA, CO, FL, IA, ID, IL, KS, KY, MA, MI, MN, ND, NE, NJ, NV, OK, OR, RI, SC, SD, TN, TX, UT, VT, WI) and Washington D.C., USA	State health departments reported to authors	Jan 2018 – Feb 2020	Ratio of Jan – Feb 2020 monthly vaccinations as a percent of Jan – Feb 2018 and Jan – Feb 2019 monthly vaccinations as a percent of Jan – Feb 2018	Monthly; Mar – Apr 2020	Ratio of 2020 monthly vaccinations as a percent of the same month of 2018 and 2019 monthly vaccinations as a percent of the same month of 2018
Virginia Mercury	2	–	DTP<br>MMR	0 – 11	VA, USA	Electronic immunization registry	Mar 2 – 6, 2020	Weekly doses administered	Weekly; Mar 9 – Apr 10, 2020	Weekly doses administered
WHO Regional Offices	1402	230,000,000 doses administered	DTP3<br>MCV1	0 – 11<br>12 – 23<br>(country-specific)	98 countries	National governments reported to WHO regional offices	Jan 2019 – Feb 2020	Ratio of doses administered in Jan – Feb 2020 and doses administered in Jan – Feb 2019	Monthly; Mar – Dec 2020	Ratio of monthly doses administered and doses administered in the same month of 2019

AL=Alabama. AR=Arkansas. CA=California. CDR=Cumulative Disruption Ratio. CO=Colorado. DHIS-2=District Health Information Software 2. DTP=diphtheria, tetanus, and pertussis. DTP3=diphtheria, tetanus, and pertussis, third dose. FL=Florida. HMIS=Health Management Information System. IA=Iowa. ID=Idaho. IL=Illinois. KS=Kansas. KY=Kentucky. MA=Massachusetts. MCV1=measles-containing vaccine, first dose. MI=Michigan. MMR=measles, mumps, and rubella. MMR1=measles, mumps, and rubella, first dose. MN=Minnesota. MR=measles and rubella. NCIRS=National Centre for Immunisation Research and Surveillance. ND=North Dakota. NE=Nebraska. NHMIS=National Health Management Information System. NJ=New Jersey. NV=Nevada. OK=Oklahoma. OR=Oregon. PCV3=pneumococcal conjugate vaccine, third dose. Penta3=pentavalent vaccine, third dose; covers diphtheria, tetanus, pertussis, hepatitis B, and Haemophilus influenzae type b. RI=Rhode Island. SC=South Carolina. SD=South Dakota. TN=Tennessee. TX=Texas. USA=United States of America. UT=Utah. VA=Virginia. VT=Vermont. WI=Wisconsin.

Supplementary table 2. Expected DTP3 and MCV1 coverage in the absence of COVID-19 and estimates of disruptions and coverage attributable to the pandemic in 2020, by country

	DTP3			MCV1		
	Expected coverage in the absence of COVID-19, % (95% UI)	Estimated relative disruption attributable to COVID-19, % (95% UI)	Estimated coverage after accounting for COVID-19 disruption, % (95% UI)	Expected coverage in the absence of COVID-19, % (95% UI)	Estimated relative disruption attributable to COVID-19, % (95% UI)	Estimated coverage after accounting for COVID-19 disruption, % (95% UI)
Afghanistan	75.2 (68.7, 81.4)	1.3 (1.0, 1.6)	74.2 (67.7, 80.4)	71.3 (59.7, 80.4)	0.3 (0.2, 0.4)	71.1 (59.6, 80.1)
Albania	99.8 (99.6, 100.0)	7.5 (0.1, 31.9)	92.4 (68.0, 99.9)	99.0 (97.2, 99.7)	8.4 (0.1, 34.5)	90.6 (64.8, 99.5)
Algeria	91.3 (86.3, 94.6)	12.9 (0.1, 45.3)	79.5 (49.8, 93.3)	83.5 (71.8, 91.9)	23.9 (0.1, 74.5)	63.5 (21.1, 89.6)
American Samoa	95.8 (87.7, 99.0)	2.1 (0.0, 14.5)	93.8 (79.2, 98.5)	96.9 (90.5, 99.5)	3.3 (0.0, 21.0)	93.8 (76.4, 99.1)
Andorra	98.8 (97.4, 99.5)	5.2 (-1.1, 28.0)	93.6 (71.5, 100.2)	99.7 (98.9, 99.9)	8.2 (0.0, 48.9)	91.5 (51.0, 99.9)
Angola	41.9 (33.6, 50.2)	0.7 (0.3, 1.1)	41.6 (33.4, 49.9)	56.4 (42.4, 70.0)	4.2 (3.0, 5.3)	54.0 (40.6, 66.9)
Antigua and Barbuda	94.1 (90.1, 96.7)	10.1 (0.1, 23.8)	84.6 (71.6, 95.4)	94.5 (88.5, 97.9)	0.0 (0.0, 0.2)	94.5 (88.5, 97.8)
Argentina	88.4 (81.9, 93.3)	7.3 (0.0, 36.4)	81.9 (56.0, 92.0)	93.7 (86.7, 97.7)	10.8 (0.0, 54.3)	83.6 (42.0, 96.8)
Armenia	92.1 (87.5, 95.3)	6.4 (0.1, 29.3)	86.2 (64.9, 94.7)	93.1 (87.5, 96.7)	7.3 (0.1, 33.1)	86.3 (62.6, 95.8)
Australia	93.2 (89.1, 96.0)	0.2 (0.0, 0.9)	93.0 (88.9, 95.9)	99.5 (98.4, 99.9)	0.9 (0.1, 4.0)	98.6 (95.3, 99.8)
Austria	88.9 (82.3, 93.6)	4.7 (0.0, 27.9)	84.7 (63.8, 92.9)	95.7 (88.8, 99.0)	7.3 (0.0, 45.2)	88.7 (53.3, 98.4)
Azerbaijan	81.7 (74.9, 87.4)	6.4 (0.1, 29.3)	76.5 (55.8, 86.7)	88.2 (82.4, 92.6)	7.3 (0.1, 33.1)	81.8 (58.9, 91.5)
Bahamas	88.2 (81.5, 93.1)	5.6 (4.5, 6.8)	83.3 (76.9, 87.9)	88.3 (78.6, 94.3)	15.3 (13.6, 17.1)	74.8 (66.3, 80.1)
Bahrain	94.6 (91.9, 96.5)	13.3 (0.1, 46.2)	81.9 (51.5, 95.6)	99.9 (99.6, 100.0)	24.9 (0.1, 75.0)	75.0 (25.0, 99.8)
Bangladesh	95.2 (91.8, 97.4)	9.9 (0.8, 19.3)	85.8 (76.3, 94.6)	93.5 (86.7, 97.4)	1.4 (0.0, 3.5)	92.1 (85.2, 96.5)
Barbados	91.2 (85.5, 95.1)	13.1 (3.8, 22.0)	79.3 (69.8, 89.2)	98.6 (96.9, 99.4)	3.4 (0.1, 15.2)	95.2 (83.2, 99.2)
Belarus	97.8 (95.4, 99.1)	5.9 (0.1, 29.2)	92.0 (68.8, 98.8)	98.0 (95.2, 99.4)	6.5 (0.1, 33.3)	91.7 (65.6, 99.1)
Belgium	98.3 (96.3, 99.4)	6.0 (-2.3, 31.2)	92.4 (67.8, 101.0)	97.4 (92.9, 99.3)	9.3 (0.0, 51.8)	88.4 (46.9, 99.2)
Belize	83.7 (78.1, 88.3)	10.1 (7.8, 12.3)	75.3 (70.0, 79.7)	89.8 (81.6, 94.9)	4.5 (2.8, 6.2)	85.8 (77.6, 91.0)

Supplementary table 2. Expected DTP3 and MCV1 coverage in the absence of COVID-19 and estimates of disruptions and coverage attributable to the pandemic in 2020, by country

	DTP3			MCV1		
	Expected coverage in the absence of COVID-19, % (95% UI)	Estimated relative disruption attributable to COVID-19, % (95% UI)	Estimated coverage after accounting for COVID-19 disruption, % (95% UI)	Expected coverage in the absence of COVID-19, % (95% UI)	Estimated relative disruption attributable to COVID-19, % (95% UI)	Estimated coverage after accounting for COVID-19 disruption, % (95% UI)
Benin	82.8 (74.6, 88.7)	4.5 (3.9, 5.1)	79.0 (71.0, 84.8)	77.1 (65.4, 86.4)	10.7 (10.0, 11.5)	68.8 (58.5, 77.0)
Bermuda	99.2 (97.9, 99.8)	22.4 (0.1, 42.1)	76.9 (57.4, 98.9)	99.8 (99.4, 100.0)	26.5 (12.6, 38.7)	73.3 (61.2, 87.3)
Bhutan	99.9 (99.6, 100.0)	0.0 (0.0, 0.1)	99.8 (99.6, 100.0)	99.6 (98.7, 99.9)	0.0 (0.0, 0.2)	99.6 (98.7, 99.9)
Bolivia (Plurinational State of)	86.6 (78.6, 92.7)	5.9 (4.8, 7.1)	81.5 (73.9, 87.3)	89.2 (79.8, 95.0)	1.2 (0.0, 2.4)	88.1 (78.9, 93.9)
Bosnia and Herzegovina	78.1 (67.6, 85.9)	6.5 (0.1, 29.9)	73.0 (53.4, 84.5)	68.9 (51.5, 83.3)	7.4 (0.1, 33.9)	63.8 (38.9, 79.9)
Botswana	79.9 (72.4, 86.0)	0.1 (0.0, 0.5)	79.8 (72.3, 86.0)	89.6 (79.1, 95.6)	0.4 (0.0, 1.1)	89.2 (78.9, 95.2)
Brazil	83.6 (77.4, 88.8)	4.0 (0.0, 16.4)	80.3 (69.0, 87.9)	99.9 (99.6, 100.0)	7.1 (0.0, 28.0)	92.8 (71.9, 99.9)
Brunei Darussalam	98.0 (96.3, 99.1)	4.5 (0.0, 25.1)	93.6 (74.1, 99.0)	99.1 (97.4, 99.8)	6.7 (0.0, 44.4)	92.4 (55.2, 99.7)
Bulgaria	92.2 (87.5, 95.7)	6.3 (0.1, 28.8)	86.4 (64.8, 94.9)	94.3 (88.0, 98.0)	7.1 (0.1, 32.7)	87.6 (63.5, 97.1)
Burkina Faso	93.2 (88.2, 96.4)	4.1 (0.1, 20.9)	89.4 (74.0, 95.7)	95.6 (88.7, 98.8)	7.2 (0.1, 35.6)	88.7 (61.5, 97.9)
Burundi	95.9 (92.6, 97.9)	4.0 (3.3, 4.7)	92.0 (88.7, 94.1)	91.3 (84.1, 96.1)	6.4 (5.3, 7.5)	85.4 (78.5, 90.1)
Cabo Verde	99.6 (99.1, 99.9)	0.0 (0.0, 0.1)	99.6 (99.1, 99.9)	99.7 (99.1, 99.9)	0.8 (0.0, 2.4)	99.0 (97.1, 99.9)
Cambodia	91.4 (85.0, 95.6)	3.2 (0.0, 23.5)	88.4 (69.0, 94.9)	91.5 (81.9, 96.8)	5.2 (0.0, 35.0)	86.7 (58.9, 95.8)
Cameroon	74.8 (65.8, 81.5)	8.3 (7.7, 8.9)	68.5 (60.3, 75.0)	68.8 (55.9, 80.1)	11.0 (10.3, 11.7)	61.2 (49.8, 71.4)
Canada	87.7 (82.5, 92.2)	5.9 (0.0, 29.5)	82.5 (62.1, 91.1)	90.7 (83.3, 95.6)	9.0 (0.0, 51.7)	82.5 (44.7, 94.4)
Central African Republic	50.0 (39.9, 60.2)	6.7 (4.0, 9.3)	46.7 (36.7, 55.9)	52.4 (36.7, 68.4)	6.3 (3.6, 9.0)	49.1 (34.4, 64.5)
Chad	41.3 (33.3, 49.5)	6.6 (0.1, 21.3)	38.5 (28.7, 48.0)	43.2 (29.3, 58.4)	12.1 (0.2, 37.3)	37.9 (22.7, 53.3)
Chile	95.4 (92.0, 97.7)	1.6 (1.1, 2.1)	93.9 (90.5, 96.1)	95.2 (90.2, 98.3)	7.1 (6.0, 8.2)	88.5 (83.5, 91.6)
China	97.0 (94.8, 98.4)	2.9 (0.0, 20.9)	94.2 (76.6, 98.1)	96.4 (92.7, 98.6)	4.6 (0.0, 32.5)	92.0 (65.1, 98.1)

Supplementary table 2. Expected DTP3 and MCV1 coverage in the absence of COVID-19 and estimates of disruptions and coverage attributable to the pandemic in 2020, by country

	DTP3			MCV1		
	Expected coverage in the absence of COVID-19, % (95% UI)	Estimated relative disruption attributable to COVID-19, % (95% UI)	Estimated coverage after accounting for COVID-19 disruption, % (95% UI)	Expected coverage in the absence of COVID-19, % (95% UI)	Estimated relative disruption attributable to COVID-19, % (95% UI)	Estimated coverage after accounting for COVID-19 disruption, % (95% UI)
Colombia	91.2 (86.2, 95.2)	5.3 (4.3, 6.3)	86.4 (81.4, 90.3)	92.8 (86.6, 96.5)	3.9 (3.1, 4.8)	89.2 (83.2, 92.8)
Comoros	84.7 (76.1, 91.2)	5.6 (1.9, 9.1)	80.0 (71.4, 87.0)	91.4 (81.9, 96.8)	12.5 (9.6, 15.5)	80.0 (71.3, 85.7)
Congo	65.5 (56.2, 73.7)	6.5 (5.4, 7.4)	61.3 (52.5, 68.8)	74.2 (60.8, 84.3)	9.9 (8.7, 11.0)	66.9 (54.9, 76.1)
Cook Islands	97.4 (94.2, 99.0)	2.1 (0.0, 14.5)	95.3 (83.5, 98.7)	99.4 (98.2, 99.9)	3.3 (0.0, 21.0)	96.1 (78.5, 99.7)
Costa Rica	97.3 (94.4, 98.8)	4.5 (-6.6, 20.4)	92.9 (77.3, 104.2)	99.8 (99.5, 100.0)	28.9 (26.9, 31.1)	71.0 (68.8, 73.0)
Croatia	93.8 (89.8, 96.5)	6.4 (0.1, 29.2)	87.8 (65.8, 96.0)	93.6 (86.8, 97.2)	7.3 (0.1, 33.3)	86.7 (62.7, 96.5)
Cuba	95.6 (92.6, 97.7)	4.4 (0.0, 16.8)	91.4 (80.3, 97.0)	90.2 (84.5, 94.3)	7.5 (0.0, 29.6)	83.5 (62.6, 92.7)
Cyprus	95.5 (92.3, 97.7)	5.2 (-1.1, 28.0)	90.6 (68.8, 97.8)	84.4 (74.8, 91.8)	8.2 (0.0, 48.9)	77.4 (42.2, 90.5)
Czechia	96.7 (93.4, 98.7)	7.4 (0.1, 31.5)	89.6 (66.4, 98.1)	95.5 (90.5, 98.4)	8.3 (0.1, 34.7)	87.6 (63.2, 97.7)
CÃ´te d'Ivoire	78.7 (71.3, 85.1)	4.8 (4.4, 5.2)	74.9 (68.1, 80.9)	76.5 (63.9, 86.1)	6.2 (5.8, 6.6)	71.7 (60.0, 80.6)
Democratic People's Republic of Korea	99.8 (99.5, 99.9)	3.8 (0.2, 9.0)	96.0 (90.8, 99.6)	99.5 (98.2, 99.9)	4.8 (0.3, 11.2)	94.7 (88.2, 99.1)
Democratic Republic of the Congo	55.0 (46.3, 63.9)	0.1 (0.0, 0.3)	55.0 (46.3, 63.9)	70.0 (58.3, 80.0)	0.1 (0.0, 0.4)	69.9 (58.2, 79.9)
Denmark	96.9 (94.5, 98.5)	4.4 (0.0, 24.7)	92.6 (71.6, 98.2)	96.6 (92.3, 98.8)	6.7 (0.0, 44.4)	90.1 (53.7, 98.5)
Djibouti	58.6 (46.4, 69.1)	6.5 (0.1, 22.4)	54.8 (39.4, 67.6)	85.4 (73.8, 92.9)	11.9 (0.2, 36.5)	75.3 (53.1, 90.6)
Dominica	97.1 (94.9, 98.5)	0.0 (0.0, 0.2)	97.0 (94.8, 98.4)	91.9 (84.9, 96.2)	0.0 (0.0, 0.2)	91.8 (84.9, 96.2)
Dominican Republic	74.6 (65.8, 82.3)	10.6 (9.4, 11.7)	66.7 (59.0, 73.6)	86.1 (76.6, 92.9)	10.0 (8.8, 11.3)	77.4 (68.9, 83.7)
Ecuador	80.8 (73.4, 87.3)	6.7 (6.1, 7.2)	75.4 (68.6, 81.5)	76.3 (65.1, 85.3)	15.9 (14.9, 17.0)	64.2 (54.5, 71.9)
Egypt	96.3 (92.9, 98.3)	14.6 (0.1, 48.5)	82.2 (49.9, 97.5)	98.6 (96.2, 99.7)	26.2 (0.1, 76.1)	72.9 (23.7, 99.2)
El Salvador	85.0 (78.2, 90.4)	27.5 (21.0, 33.4)	61.6 (54.6, 68.7)	92.3 (86.1, 96.4)	24.9 (19.7, 30.3)	69.3 (62.7, 75.1)

Supplementary table 2. Expected DTP3 and MCV1 coverage in the absence of COVID-19 and estimates of disruptions and coverage attributable to the pandemic in 2020, by country

	DTP3			MCV1		
	Expected coverage in the absence of COVID-19, % (95% UI)	Estimated relative disruption attributable to COVID-19, % (95% UI)	Estimated coverage after accounting for COVID-19 disruption, % (95% UI)	Expected coverage in the absence of COVID-19, % (95% UI)	Estimated relative disruption attributable to COVID-19, % (95% UI)	Estimated coverage after accounting for COVID-19 disruption, % (95% UI)
Equatorial Guinea	54.7 (39.5, 68.3)	2.9 (0.4, 5.5)	53.1 (38.1, 66.4)	45.2 (29.3, 62.8)	-0.1 (-5.8, 3.4)	45.2 (29.2, 62.9)
Eritrea	91.7 (85.9, 95.6)	0.0 (0.0, 0.0)	91.7 (85.9, 95.6)	99.6 (98.9, 99.9)	0.2 (0.0, 0.7)	99.5 (98.5, 99.9)
Estonia	92.3 (87.6, 95.6)	6.4 (0.1, 29.2)	86.4 (65.0, 94.8)	89.6 (81.2, 94.7)	7.3 (0.1, 33.2)	83.1 (59.5, 93.7)
Eswatini	87.6 (81.3, 92.3)	5.3 (2.1, 8.5)	82.9 (76.6, 88.0)	88.8 (80.2, 94.3)	23.5 (20.2, 26.9)	67.9 (61.1, 73.1)
Ethiopia	57.6 (49.1, 65.7)	3.8 (3.2, 4.5)	55.4 (47.2, 63.3)	61.3 (50.2, 72.5)	1.0 (0.6, 1.4)	60.6 (49.5, 71.9)
Fiji	97.1 (94.0, 98.8)	2.2 (0.0, 15.0)	95.0 (82.1, 98.5)	90.9 (81.1, 96.3)	3.4 (0.0, 21.8)	87.9 (70.6, 95.4)
Finland	90.7 (85.3, 94.5)	4.0 (0.0, 21.6)	87.1 (70.2, 93.7)	96.7 (92.3, 98.8)	5.7 (0.0, 37.8)	91.2 (59.8, 98.7)
France	96.4 (93.1, 98.3)	6.2 (-1.4, 31.4)	90.4 (65.9, 98.1)	91.3 (82.2, 96.6)	9.4 (0.0, 52.6)	82.7 (43.6, 95.5)
Gabon	74.5 (63.9, 82.9)	5.2 (3.6, 6.7)	70.6 (60.6, 78.8)	70.6 (57.1, 82.3)	17.1 (15.1, 19.3)	58.5 (46.9, 68.2)
Gambia	91.3 (86.2, 94.8)	12.2 (11.2, 13.1)	80.2 (75.8, 83.3)	85.7 (76.4, 92.0)	10.1 (8.9, 11.3)	77.0 (68.4, 82.8)
Georgia	86.4 (79.0, 92.1)	8.5 (0.1, 34.0)	79.0 (57.0, 91.0)	81.2 (71.9, 88.1)	9.8 (0.1, 38.0)	73.3 (50.4, 86.7)
Germany	96.8 (92.4, 99.1)	5.0 (0.0, 28.1)	91.9 (69.4, 98.7)	98.4 (94.8, 99.7)	7.6 (0.0, 46.9)	90.9 (52.0, 99.5)
Ghana	95.1 (91.4, 97.5)	0.5 (0.1, 1.2)	94.7 (91.1, 97.1)	92.8 (85.3, 97.2)	0.6 (0.0, 1.2)	92.3 (84.8, 96.5)
Greece	99.9 (99.6, 100.0)	5.8 (-1.3, 29.2)	94.1 (70.7, 101.2)	98.2 (94.8, 99.6)	8.9 (0.0, 49.7)	89.4 (49.8, 99.4)
Greenland	96.2 (93.1, 98.1)	5.4 (0.0, 28.4)	91.1 (69.5, 97.4)	97.0 (92.7, 99.0)	8.2 (0.0, 48.5)	89.0 (49.7, 98.7)
Grenada	89.9 (83.6, 94.5)	3.3 (0.1, 14.6)	87.0 (76.1, 93.6)	96.0 (92.3, 98.4)	3.6 (0.6, 6.8)	92.5 (87.9, 96.5)
Guam	98.5 (95.8, 99.6)	2.4 (0.0, 17.0)	96.1 (81.7, 99.4)	99.1 (97.2, 99.8)	3.7 (0.0, 24.8)	95.4 (74.3, 99.7)
Guatemala	78.7 (70.7, 85.4)	4.9 (1.9, 7.7)	74.8 (67.4, 81.3)	85.1 (74.7, 92.0)	4.8 (3.3, 6.5)	81.0 (71.2, 87.7)
Guinea	47.7 (38.0, 58.1)	24.1 (22.8, 25.3)	36.2 (28.9, 44.0)	51.4 (38.4, 65.4)	25.9 (24.7, 27.1)	38.1 (28.3, 48.2)

Supplementary table 2. Expected DTP3 and MCV1 coverage in the absence of COVID-19 and estimates of disruptions and coverage attributable to the pandemic in 2020, by country

	DTP3			MCV1		
	Expected coverage in the absence of COVID-19, % (95% UI)	Estimated relative disruption attributable to COVID-19, % (95% UI)	Estimated coverage after accounting for COVID-19 disruption, % (95% UI)	Expected coverage in the absence of COVID-19, % (95% UI)	Estimated relative disruption attributable to COVID-19, % (95% UI)	Estimated coverage after accounting for COVID-19 disruption, % (95% UI)
Guinea-Bissau	73.4 (63.7, 81.1)	6.6 (0.1, 21.3)	68.5 (55.5, 79.2)	79.4 (65.8, 89.0)	12.1 (0.2, 37.3)	69.8 (47.4, 85.8)
Guyana	92.0 (88.2, 94.7)	7.8 (0.2, 23.5)	84.8 (70.1, 93.8)	94.7 (90.1, 97.5)	8.4 (0.1, 17.0)	86.8 (77.8, 95.5)
Haiti	60.0 (50.1, 69.3)	2.8 (1.4, 4.1)	58.3 (48.8, 67.3)	72.6 (57.0, 86.1)	32.8 (30.2, 35.3)	48.8 (38.0, 58.0)
Honduras	86.5 (80.4, 91.2)	1.9 (1.3, 2.6)	84.9 (78.8, 89.5)	90.4 (83.8, 95.3)	3.3 (2.2, 4.4)	87.4 (80.9, 92.2)
Hungary	99.3 (98.3, 99.8)	5.8 (0.1, 30.5)	93.5 (69.0, 99.6)	99.9 (99.6, 100.0)	6.2 (0.1, 32.8)	93.7 (66.9, 99.9)
Iceland	90.8 (85.0, 94.9)	5.2 (-1.1, 28.0)	86.1 (64.1, 94.2)	93.8 (86.6, 97.7)	8.2 (0.0, 48.9)	86.1 (47.8, 96.9)
India	88.1 (82.7, 92.0)	15.8 (13.2, 18.6)	74.2 (69.1, 78.6)	95.1 (89.4, 98.1)	9.8 (8.0, 11.9)	85.7 (80.5, 89.1)
Indonesia	78.2 (71.0, 84.2)	16.5 (8.7, 26.5)	65.4 (55.3, 74.1)	81.7 (71.2, 89.9)	15.1 (8.2, 23.8)	69.3 (59.1, 78.9)
Iran (Islamic Republic of)	98.0 (96.5, 99.0)	12.9 (0.1, 45.3)	85.4 (53.8, 98.5)	99.2 (97.6, 99.8)	23.9 (0.1, 74.5)	75.5 (25.1, 99.5)
Iraq	76.4 (67.7, 83.1)	9.0 (8.5, 9.4)	69.6 (61.8, 75.7)	81.1 (69.4, 90.0)	7.1 (6.6, 7.6)	75.3 (64.3, 83.7)
Ireland	94.7 (91.2, 97.0)	7.5 (0.0, 38.0)	87.5 (59.4, 96.4)	92.7 (84.2, 97.1)	11.0 (0.0, 55.0)	82.4 (41.2, 96.2)
Israel	97.4 (95.0, 98.8)	5.4 (-2.7, 28.6)	92.1 (68.8, 99.9)	99.2 (97.7, 99.9)	8.7 (0.0, 49.8)	90.6 (50.0, 99.8)
Italy	99.7 (99.3, 99.9)	5.4 (0.0, 27.0)	94.3 (72.9, 99.9)	88.0 (79.9, 93.2)	8.6 (0.0, 46.7)	80.4 (48.0, 92.2)
Jamaica	95.5 (91.8, 97.8)	5.5 (0.3, 10.0)	90.2 (84.7, 95.6)	99.8 (99.5, 100.0)	0.4 (0.0, 2.2)	99.4 (97.6, 99.9)
Japan	98.4 (96.2, 99.4)	4.5 (0.0, 24.7)	94.0 (74.0, 99.3)	98.0 (94.9, 99.5)	6.7 (0.0, 44.4)	91.5 (54.5, 99.2)
Jordan	86.6 (81.8, 90.4)	10.6 (-0.3, 39.7)	77.4 (52.6, 89.6)	79.3 (69.4, 86.9)	20.5 (-2.8, 71.0)	63.0 (22.2, 85.0)
Kazakhstan	94.8 (91.5, 97.1)	6.2 (0.1, 29.5)	88.9 (67.0, 96.6)	92.1 (88.0, 95.1)	6.9 (0.1, 32.7)	85.8 (61.7, 94.4)
Kenya	87.8 (81.3, 92.7)	0.2 (0.1, 0.5)	87.6 (81.3, 92.6)	84.9 (72.9, 92.7)	0.2 (0.1, 0.6)	84.7 (72.6, 92.5)
Kiribati	52.5 (43.9, 60.4)	2.1 (0.0, 14.5)	51.4 (41.5, 59.5)	64.5 (49.5, 76.9)	3.3 (0.0, 21.0)	62.4 (45.4, 75.5)

Supplementary table 2. Expected DTP3 and MCV1 coverage in the absence of COVID-19 and estimates of disruptions and coverage attributable to the pandemic in 2020, by country

	DTP3			MCV1		
	Expected coverage in the absence of COVID-19, % (95% UI)	Estimated relative disruption attributable to COVID-19, % (95% UI)	Estimated coverage after accounting for COVID-19 disruption, % (95% UI)	Expected coverage in the absence of COVID-19, % (95% UI)	Estimated relative disruption attributable to COVID-19, % (95% UI)	Estimated coverage after accounting for COVID-19 disruption, % (95% UI)
Kuwait	94.4 (89.8, 97.3)	15.5 (0.1, 48.9)	79.8 (48.2, 96.4)	95.9 (91.2, 98.5)	25.7 (0.0, 74.8)	71.2 (23.7, 98.0)
Kyrgyzstan	88.3 (83.0, 92.1)	9.7 (-0.7, 36.3)	79.7 (56.2, 91.7)	89.6 (83.5, 94.1)	11.0 (-0.5, 42.9)	79.7 (49.8, 92.8)
Lao People's Democratic Republic	65.0 (57.0, 72.5)	3.9 (0.0, 11.9)	62.4 (52.8, 70.8)	75.1 (61.9, 85.7)	4.0 (0.0, 10.6)	72.0 (59.2, 83.9)
Latvia	99.9 (99.7, 100.0)	6.5 (0.1, 30.0)	93.4 (69.9, 99.9)	99.6 (98.8, 99.9)	7.4 (0.1, 34.2)	92.2 (65.7, 99.8)
Lebanon	85.7 (80.7, 89.8)	11.7 (0.1, 42.4)	75.7 (49.2, 88.8)	79.9 (70.6, 87.6)	22.5 (0.1, 72.6)	61.9 (21.2, 84.9)
Lesotho	86.1 (79.0, 91.7)	0.7 (0.0, 2.1)	85.5 (78.2, 91.1)	90.8 (82.8, 96.1)	0.5 (0.0, 1.7)	90.3 (82.4, 95.7)
Liberia	89.2 (81.0, 94.9)	1.3 (0.4, 2.1)	88.1 (80.0, 93.9)	93.2 (83.6, 97.9)	1.5 (0.9, 2.3)	91.8 (82.5, 96.5)
Libya	85.1 (80.9, 88.8)	9.7 (0.1, 37.2)	76.9 (53.5, 87.9)	88.7 (83.0, 93.1)	19.4 (0.1, 68.7)	71.5 (26.9, 91.7)
Lithuania	92.8 (87.9, 96.1)	8.7 (0.1, 35.5)	84.7 (59.9, 94.9)	94.4 (88.8, 97.8)	10.0 (0.1, 38.2)	85.0 (58.2, 96.7)
Luxembourg	99.2 (98.4, 99.7)	5.5 (-3.2, 29.3)	93.7 (70.1, 102.2)	98.6 (96.6, 99.5)	8.7 (-0.7, 48.3)	90.0 (51.1, 99.6)
Madagascar	61.9 (54.3, 69.2)	1.4 (1.2, 1.6)	61.0 (53.6, 68.2)	62.0 (49.2, 73.8)	0.1 (0.1, 0.4)	61.9 (49.2, 73.7)
Malawi	94.2 (90.0, 96.9)	0.0 (0.0, 0.0)	94.2 (90.0, 96.9)	96.6 (92.0, 98.9)	0.0 (0.0, 0.2)	96.5 (91.9, 98.8)
Malaysia	97.9 (95.7, 99.0)	0.3 (0.0, 1.2)	97.6 (95.3, 98.9)	99.5 (98.6, 99.9)	0.2 (0.0, 1.1)	99.3 (98.2, 99.9)
Maldives	87.2 (82.8, 90.9)	0.8 (0.0, 3.8)	86.5 (81.4, 90.5)	91.9 (85.8, 95.9)	3.2 (1.0, 6.7)	89.0 (82.9, 93.7)
Mali	75.0 (67.7, 81.4)	10.3 (9.9, 10.7)	67.3 (60.7, 73.0)	72.3 (60.2, 83.3)	10.3 (10.0, 10.7)	64.8 (54.0, 74.6)
Malta	97.2 (94.3, 98.9)	4.3 (-1.5, 25.6)	93.1 (72.0, 99.2)	95.4 (88.2, 98.6)	6.8 (0.0, 44.6)	88.9 (52.1, 98.2)
Marshall Islands	80.5 (72.5, 87.4)	2.1 (0.0, 14.5)	78.8 (67.5, 86.7)	84.5 (72.2, 93.0)	3.3 (0.0, 21.0)	81.8 (65.2, 91.8)
Mauritania	69.3 (55.3, 81.9)	7.9 (6.7, 9.1)	63.8 (50.9, 75.4)	82.0 (71.9, 90.3)	1.7 (0.1, 3.4)	80.6 (70.7, 88.7)
Mauritius	95.3 (91.9, 97.5)	1.8 (0.4, 3.5)	93.5 (89.9, 96.2)	98.6 (96.4, 99.6)	0.0 (0.0, 0.0)	98.5 (96.4, 99.5)

Supplementary table 2. Expected DTP3 and MCV1 coverage in the absence of COVID-19 and estimates of disruptions and coverage attributable to the pandemic in 2020, by country

	DTP3			MCV1		
	**Expected coverage in the absence of COVID-19, % (95% UI)**	**Estimated relative disruption attributable to COVID-19, % (95% UI)**	**Estimated coverage after accounting for COVID-19 disruption, % (95% UI)**	**Expected coverage in the absence of COVID-19, % (95% UI)**	**Estimated relative disruption attributable to COVID-19, % (95% UI)**	**Estimated coverage after accounting for COVID-19 disruption, % (95% UI)**
Mexico	72.0 (64.4, 78.8)	4.4 (0.0, 16.8)	68.8 (57.8, 76.9)	84.6 (74.5, 91.9)	7.5 (0.0, 29.7)	78.3 (57.3, 89.9)
Micronesia (Federated States of)	76.3 (66.6, 83.9)	2.1 (0.0, 14.5)	74.6 (62.7, 82.9)	76.6 (62.7, 86.6)	3.3 (0.0, 21.0)	74.0 (58.5, 85.4)
Monaco	99.2 (97.9, 99.8)	5.2 (-1.1, 28.0)	94.0 (71.4, 100.4)	89.3 (80.9, 94.6)	8.2 (0.0, 48.9)	81.9 (46.1, 93.8)
Mongolia	80.8 (75.6, 85.1)	0.1 (0.0, 0.6)	80.7 (75.5, 85.1)	83.2 (75.6, 89.4)	0.1 (0.0, 0.5)	83.2 (75.4, 89.3)
Montenegro	85.6 (78.3, 91.2)	7.5 (0.1, 31.9)	79.2 (57.4, 89.9)	63.9 (46.1, 79.3)	8.4 (0.1, 34.5)	58.5 (37.7, 76.3)
Morocco	94.5 (90.1, 97.4)	19.3 (0.1, 59.9)	76.3 (37.4, 96.8)	93.1 (85.0, 97.3)	30.4 (-0.7, 76.9)	64.8 (21.6, 96.0)
Mozambique	95.5 (92.1, 97.7)	6.7 (6.5, 6.9)	89.1 (85.9, 91.1)	96.5 (91.2, 98.9)	2.9 (2.7, 3.0)	93.7 (88.6, 96.1)
Myanmar	73.4 (65.6, 79.9)	2.0 (0.9, 3.7)	72.0 (64.0, 78.5)	83.0 (72.9, 90.2)	11.3 (6.1, 19.1)	73.6 (62.4, 82.0)
Namibia	90.0 (82.5, 95.0)	0.3 (0.0, 1.3)	89.7 (82.1, 94.8)	99.7 (99.0, 99.9)	0.2 (0.0, 1.1)	99.4 (98.5, 99.9)
Nauru	99.4 (98.6, 99.8)	2.1 (0.0, 14.5)	97.3 (85.0, 99.7)	99.7 (99.0, 99.9)	3.3 (0.0, 21.0)	96.4 (79.0, 99.9)
Nepal	86.8 (81.3, 91.3)	6.7 (6.4, 6.9)	81.1 (76.0, 85.2)	96.7 (92.0, 99.0)	3.0 (2.6, 3.4)	93.8 (89.2, 96.0)
Netherlands	94.0 (90.2, 96.7)	5.9 (0.0, 30.0)	88.4 (66.2, 96.0)	94.7 (88.6, 97.9)	9.0 (0.0, 51.7)	86.2 (45.8, 97.1)
New Zealand	92.9 (88.4, 96.3)	3.6 (-3.6, 23.0)	89.6 (71.9, 97.2)	93.4 (86.3, 97.5)	5.6 (-1.0, 37.7)	88.2 (58.8, 97.1)
Nicaragua	99.9 (99.6, 100.0)	3.2 (0.0, 15.4)	96.7 (84.5, 99.9)	99.9 (99.5, 100.0)	5.6 (0.0, 25.1)	94.3 (74.8, 99.9)
Niger	60.7 (51.8, 69.7)	4.3 (3.6, 5.0)	58.0 (49.6, 66.6)	64.8 (49.6, 78.0)	0.9 (0.2, 1.7)	64.2 (49.0, 77.2)
Nigeria	60.0 (49.4, 69.5)	4.0 (3.6, 4.5)	57.6 (47.5, 66.7)	62.2 (45.5, 77.2)	6.1 (5.6, 6.5)	58.4 (42.7, 72.4)
Niue	98.0 (96.1, 99.1)	2.1 (0.0, 14.5)	95.9 (83.5, 98.9)	99.9 (99.5, 100.0)	3.3 (0.0, 21.0)	96.6 (79.0, 99.9)
North Macedonia	87.1 (80.7, 91.8)	9.4 (-0.1, 34.8)	78.9 (56.5, 91.1)	78.6 (64.5, 89.1)	10.7 (-0.1, 41.3)	70.2 (42.4, 86.8)
Northern Mariana Islands	98.3 (95.4, 99.6)	3.5 (0.0, 26.2)	94.9 (72.6, 99.3)	99.0 (96.6, 99.8)	5.6 (0.0, 34.5)	93.4 (64.4, 99.6)

Supplementary table 2. Expected DTP3 and MCV1 coverage in the absence of COVID-19 and estimates of disruptions and coverage attributable to the pandemic in 2020, by country

	DTP3			MCV1		
	Expected coverage in the absence of COVID-19, % (95% UI)	Estimated relative disruption attributable to COVID-19, % (95% UI)	Estimated coverage after accounting for COVID-19 disruption, % (95% UI)	Expected coverage in the absence of COVID-19, % (95% UI)	Estimated relative disruption attributable to COVID-19, % (95% UI)	Estimated coverage after accounting for COVID-19 disruption, % (95% UI)
Norway	96.5 (93.8, 98.3)	4.9 (0.0, 28.5)	91.8 (69.1, 97.9)	97.6 (94.7, 99.3)	7.5 (0.0, 46.7)	90.3 (52.5, 99.0)
Oman	99.0 (97.8, 99.6)	13.8 (0.1, 47.2)	85.3 (52.3, 99.3)	99.9 (99.6, 100.0)	25.3 (0.1, 75.5)	74.6 (24.5, 99.8)
Pakistan	84.9 (77.1, 90.4)	5.2 (4.8, 5.5)	80.5 (73.1, 85.8)	78.1 (65.0, 88.5)	1.8 (1.4, 2.2)	76.7 (63.8, 86.9)
Palau	94.3 (89.7, 97.2)	2.1 (0.0, 14.5)	92.3 (80.6, 96.8)	93.9 (86.8, 98.0)	3.3 (0.0, 21.0)	90.9 (73.6, 97.6)
Palestine	99.0 (97.5, 99.6)	12.9 (0.1, 45.3)	86.2 (54.3, 99.3)	99.5 (98.5, 99.9)	23.9 (0.1, 74.5)	75.7 (25.4, 99.7)
Panama	83.6 (77.0, 88.8)	20.1 (18.9, 21.4)	66.8 (61.6, 71.2)	93.3 (88.4, 96.7)	17.2 (16.1, 18.5)	77.3 (72.7, 80.4)
Papua New Guinea	35.4 (26.8, 44.5)	4.1 (1.8, 7.9)	34.0 (25.7, 43.1)	45.4 (28.2, 63.6)	4.3 (2.0, 7.7)	43.5 (27.0, 60.5)
Paraguay	88.2 (81.4, 93.3)	0.6 (0.0, 1.8)	87.7 (80.8, 93.0)	94.0 (86.2, 98.0)	18.9 (14.5, 22.9)	76.2 (69.7, 81.7)
Peru	85.4 (79.7, 89.9)	31.8 (30.7, 33.0)	58.2 (54.2, 61.5)	85.8 (76.0, 92.7)	17.8 (16.9, 18.6)	70.5 (62.4, 76.3)
Philippines	83.9 (75.8, 89.8)	18.7 (9.6, 30.9)	68.2 (57.2, 78.3)	80.2 (68.6, 88.3)	16.9 (9.1, 26.6)	66.6 (55.0, 76.1)
Poland	96.6 (93.6, 98.5)	7.4 (0.1, 31.8)	89.5 (66.4, 97.9)	95.5 (89.9, 98.6)	8.4 (0.1, 34.6)	87.6 (61.6, 97.8)
Portugal	98.9 (97.7, 99.6)	5.9 (-3.3, 30.8)	93.1 (68.2, 102.4)	99.7 (99.1, 100.0)	9.1 (-0.6, 50.3)	90.7 (49.6, 100.2)
Puerto Rico	88.8 (79.1, 95.0)	4.4 (0.0, 16.9)	84.9 (70.5, 93.9)	92.1 (79.8, 98.1)	7.5 (0.0, 30.7)	85.1 (61.8, 96.8)
Qatar	97.5 (94.8, 99.0)	13.5 (0.1, 46.2)	84.4 (52.0, 98.3)	99.9 (99.6, 100.0)	24.7 (0.1, 75.1)	75.3 (24.8, 99.8)
Republic of Korea	95.9 (92.6, 98.1)	4.3 (0.0, 24.4)	91.8 (72.5, 97.7)	97.0 (92.3, 99.0)	6.0 (0.0, 39.2)	91.2 (59.2, 98.7)
Republic of Moldova	92.4 (87.8, 95.6)	8.0 (0.1, 33.1)	85.0 (62.2, 95.0)	90.3 (84.4, 94.6)	9.2 (0.1, 36.2)	82.0 (57.6, 93.8)
Romania	88.2 (82.1, 92.3)	9.6 (-1.8, 36.0)	79.7 (56.1, 91.5)	90.7 (83.1, 95.8)	11.0 (-1.3, 43.4)	80.7 (51.1, 95.0)
Russian Federation	97.1 (94.7, 98.7)	6.4 (0.1, 29.3)	90.9 (68.0, 98.3)	99.5 (98.5, 99.9)	7.2 (0.1, 32.5)	92.3 (67.3, 99.8)
Rwanda	96.4 (93.8, 98.1)	5.7 (3.5, 7.9)	90.9 (87.9, 93.5)	92.7 (87.3, 96.1)	2.6 (0.3, 8.4)	90.3 (83.0, 95.4)

Supplementary table 2. Expected DTP3 and MCV1 coverage in the absence of COVID-19 and estimates of disruptions and coverage attributable to the pandemic in 2020, by country

	DTP3			MCV1		
	**Expected coverage in the absence of COVID-19, % (95% UI)**	**Estimated relative disruption attributable to COVID-19, % (95% UI)**	**Estimated coverage after accounting for COVID-19 disruption, % (95% UI)**	**Expected coverage in the absence of COVID-19, % (95% UI)**	**Estimated relative disruption attributable to COVID-19, % (95% UI)**	**Estimated coverage after accounting for COVID-19 disruption, % (95% UI)**
Saint Kitts and Nevis	96.1 (93.2, 98.0)	0.0 (0.0, 0.1)	96.1 (93.2, 98.0)	97.1 (92.8, 99.1)	1.0 (0.0, 4.3)	96.2 (91.3, 98.8)
Saint Lucia	88.9 (83.3, 93.4)	0.0 (0.0, 0.1)	88.9 (83.3, 93.4)	94.2 (88.4, 97.5)	1.3 (0.0, 6.2)	93.0 (86.2, 97.2)
Saint Vincent and the Grenadines	97.5 (95.3, 98.8)	2.3 (0.1, 11.2)	95.2 (86.5, 98.6)	99.8 (99.3, 100.0)	4.9 (0.1, 13.5)	94.9 (86.3, 99.8)
Samoa	53.3 (44.6, 62.6)	2.1 (0.0, 14.5)	52.2 (41.8, 61.6)	79.8 (67.0, 89.3)	3.3 (0.0, 21.0)	77.2 (61.1, 87.8)
San Marino	88.1 (82.0, 92.5)	5.2 (-1.1, 28.0)	83.6 (62.0, 92.3)	87.6 (77.9, 93.9)	8.2 (0.0, 48.9)	80.4 (46.6, 92.4)
Sao Tome and Principe	93.6 (88.8, 96.6)	8.4 (6.9, 9.7)	85.8 (80.9, 89.0)	98.8 (96.7, 99.7)	14.8 (12.4, 17.3)	84.2 (81.3, 87.0)
Saudi Arabia	94.0 (90.9, 96.2)	20.4 (0.1, 62.6)	74.8 (35.1, 95.2)	99.4 (97.9, 99.9)	32.0 (0.1, 78.2)	67.6 (21.8, 99.6)
Senegal	95.8 (93.4, 97.5)	16.5 (16.0, 17.0)	80.0 (77.9, 81.5)	89.3 (80.4, 94.8)	16.1 (15.5, 16.5)	74.9 (67.5, 79.5)
Serbia	85.6 (80.6, 89.7)	7.9 (-0.6, 32.5)	78.9 (57.9, 88.9)	95.7 (89.8, 98.5)	9.0 (-1.0, 36.0)	87.1 (61.9, 98.3)
Seychelles	97.3 (94.7, 98.7)	0.7 (0.0, 1.9)	96.6 (94.1, 98.3)	99.4 (98.4, 99.9)	5.5 (3.3, 7.8)	93.9 (91.4, 96.4)
Sierra Leone	88.0 (80.7, 93.0)	3.4 (2.8, 4.0)	85.0 (78.1, 90.0)	90.9 (80.9, 96.5)	8.0 (7.4, 8.7)	83.6 (74.5, 88.7)
Singapore	92.5 (88.2, 95.5)	4.9 (-2.2, 27.6)	88.0 (67.7, 95.7)	90.0 (81.3, 95.6)	7.8 (0.0, 46.9)	83.0 (47.0, 94.5)
Slovakia	96.8 (93.9, 98.4)	8.7 (0.1, 34.9)	88.3 (62.6, 98.2)	97.0 (93.2, 98.9)	10.0 (0.1, 38.5)	87.3 (59.4, 98.6)
Slovenia	94.4 (90.5, 96.9)	9.0 (-0.1, 34.6)	85.9 (62.5, 96.8)	94.7 (89.1, 97.7)	10.3 (0.1, 39.7)	84.9 (56.5, 97.3)
Solomon Islands	89.6 (83.0, 94.2)	2.1 (0.0, 14.5)	87.7 (75.9, 93.7)	94.8 (87.8, 98.2)	3.3 (0.0, 21.0)	91.7 (75.2, 97.4)
Somalia	17.4 (10.9, 25.6)	0.1 (0.0, 0.4)	17.3 (10.8, 25.6)	28.1 (15.9, 44.1)	0.1 (0.1, 0.4)	28.0 (15.9, 44.1)
South Africa	63.1 (54.8, 70.9)	0.2 (0.0, 0.7)	63.0 (54.7, 70.9)	78.3 (66.9, 87.3)	1.4 (0.9, 1.9)	77.2 (65.8, 86.1)
South Sudan	58.5 (45.4, 71.4)	6.5 (0.1, 22.4)	54.7 (38.7, 68.4)	48.1 (31.7, 64.4)	11.9 (0.2, 36.5)	42.4 (24.6, 61.3)
Spain	96.1 (92.8, 98.2)	7.3 (1.2, 21.9)	89.1 (74.7, 95.9)	98.9 (96.7, 99.7)	15.7 (1.3, 53.6)	83.4 (45.8, 97.5)

Supplementary table 2. Expected DTP3 and MCV1 coverage in the absence of COVID-19 and estimates of disruptions and coverage attributable to the pandemic in 2020, by country

	DTP3			MCV1		
	Expected coverage in the absence of COVID-19, % (95% UI)	Estimated relative disruption attributable to COVID-19, % (95% UI)	Estimated coverage after accounting for COVID-19 disruption, % (95% UI)	Expected coverage in the absence of COVID-19, % (95% UI)	Estimated relative disruption attributable to COVID-19, % (95% UI)	Estimated coverage after accounting for COVID-19 disruption, % (95% UI)
Sri Lanka	97.5 (95.6, 98.6)	4.6 (0.0, 29.6)	93.0 (68.6, 98.4)	92.4 (85.3, 96.6)	7.1 (0.0, 39.9)	85.8 (56.1, 95.4)
Sudan	76.1 (69.7, 81.6)	0.9 (0.7, 1.1)	75.4 (69.1, 80.9)	85.6 (75.9, 92.3)	0.6 (0.5, 0.8)	85.0 (75.4, 91.7)
Suriname	77.6 (70.7, 83.8)	7.8 (0.1, 19.4)	71.6 (60.9, 81.1)	84.1 (74.1, 91.4)	7.5 (0.1, 15.3)	77.8 (66.8, 87.8)
Sweden	97.8 (95.9, 99.0)	4.4 (0.0, 25.3)	93.4 (72.1, 98.7)	98.3 (95.7, 99.5)	6.4 (0.0, 42.5)	92.0 (55.7, 99.4)
Switzerland	96.2 (93.4, 98.0)	3.7 (0.0, 22.2)	92.6 (74.5, 97.9)	96.0 (91.2, 98.6)	5.5 (0.0, 35.6)	90.8 (61.7, 98.3)
Syrian Arab Republic	75.8 (68.3, 83.2)	0.2 (0.0, 0.6)	75.7 (68.2, 82.9)	78.2 (65.9, 87.4)	0.1 (-1.5, 1.0)	78.2 (65.8, 87.1)
Taiwan (Province of China)	97.9 (94.4, 99.5)	1.5 (0.0, 9.6)	96.4 (88.5, 99.3)	99.8 (99.3, 100.0)	2.4 (0.0, 12.5)	97.4 (87.2, 99.9)
Tajikistan	89.3 (85.5, 92.5)	6.5 (0.1, 29.8)	83.4 (61.3, 91.7)	90.7 (83.4, 95.3)	7.5 (0.1, 32.1)	83.9 (60.9, 94.1)
Thailand	87.4 (83.1, 91.0)	2.5 (0.0, 18.7)	85.3 (71.4, 90.4)	90.5 (84.6, 94.6)	3.9 (0.0, 27.5)	87.0 (66.7, 93.7)
Timor-Leste	63.2 (53.0, 73.5)	4.6 (0.0, 14.0)	60.3 (49.7, 71.0)	68.8 (55.5, 79.7)	3.6 (0.0, 11.3)	66.3 (53.2, 77.8)
Togo	80.1 (72.6, 86.2)	5.8 (0.1, 23.1)	75.4 (60.9, 84.6)	79.2 (67.5, 88.6)	10.8 (0.1, 36.4)	70.6 (50.6, 86.1)
Tokelau	96.1 (89.1, 99.1)	2.1 (0.0, 14.5)	94.0 (81.7, 98.7)	97.1 (90.7, 99.5)	3.3 (0.0, 21.0)	93.9 (77.4, 99.1)
Tonga	99.8 (99.5, 100.0)	2.1 (0.0, 14.5)	97.7 (85.5, 99.9)	92.1 (86.7, 96.0)	3.3 (0.0, 21.0)	89.1 (72.4, 95.3)
Trinidad and Tobago	77.1 (70.8, 82.3)	0.0 (0.0, 0.2)	77.1 (70.8, 82.3)	92.7 (84.9, 96.9)	3.2 (0.0, 11.4)	89.8 (80.0, 96.3)
Tunisia	95.8 (93.5, 97.5)	12.9 (0.1, 45.3)	83.5 (52.7, 96.8)	95.5 (91.2, 98.0)	23.9 (0.1, 74.5)	72.7 (24.4, 97.1)
Turkey	94.3 (90.4, 97.1)	15.7 (0.1, 49.4)	79.5 (47.8, 95.9)	90.9 (84.3, 95.6)	27.3 (0.1, 76.3)	66.1 (21.7, 94.0)
Turkmenistan	99.6 (99.1, 99.9)	6.4 (0.1, 29.3)	93.2 (70.5, 99.7)	94.3 (90.7, 96.8)	7.3 (0.1, 33.1)	87.5 (63.5, 96.0)
Tuvalu	91.5 (84.7, 96.2)	2.1 (0.0, 14.5)	89.6 (77.1, 95.6)	93.6 (86.5, 97.6)	3.3 (0.0, 21.0)	90.5 (74.2, 97.2)
Uganda	74.2 (62.4, 83.7)	0.5 (0.3, 0.8)	73.8 (62.1, 83.2)	75.2 (61.0, 85.7)	0.4 (0.1, 0.7)	74.9 (60.8, 85.4)

Supplementary table 2. Expected DTP3 and MCV1 coverage in the absence of COVID-19 and estimates of disruptions and coverage attributable to the pandemic in 2020, by country

	DTP3			MCV1		
	Expected coverage in the absence of COVID-19, % (95% UI)	Estimated relative disruption attributable to COVID-19, % (95% UI)	Estimated coverage after accounting for COVID-19 disruption, % (95% UI)	Expected coverage in the absence of COVID-19, % (95% UI)	Estimated relative disruption attributable to COVID-19, % (95% UI)	Estimated coverage after accounting for COVID-19 disruption, % (95% UI)
Ukraine	93.7 (90.3, 96.5)	6.4 (0.1, 29.2)	87.7 (66.2, 95.7)	99.1 (97.8, 99.7)	7.3 (0.1, 33.3)	91.9 (66.5, 99.5)
United Arab Emirates	94.6 (91.3, 96.9)	20.3 (0.1, 61.0)	75.4 (37.0, 95.4)	93.6 (89.1, 96.4)	32.2 (0.1, 78.4)	63.5 (20.4, 95.1)
United Kingdom	93.8 (90.0, 96.5)	0.0 (0.0, 0.0)	93.7 (90.0, 96.5)	92.6 (85.7, 96.7)	0.0 (0.0, 0.0)	92.6 (85.7, 96.6)
United Republic of Tanzania	90.2 (86.7, 92.7)	4.3 (3.9, 4.7)	86.3 (83.1, 88.7)	89.0 (83.4, 93.4)	3.8 (3.4, 4.2)	85.6 (80.1, 90.0)
United States Virgin Islands	66.4 (47.3, 82.9)	4.0 (0.0, 21.4)	63.7 (44.1, 81.4)	71.1 (40.5, 91.6)	5.7 (0.0, 27.5)	67.0 (36.5, 89.9)
United States of America	92.9 (90.0, 95.3)	9.1 (4.2, 16.1)	84.5 (77.7, 89.8)	91.7 (85.4, 96.0)	9.0 (5.2, 14.7)	83.4 (76.0, 89.3)
Uruguay	93.1 (88.4, 96.3)	0.5 (0.0, 1.8)	92.6 (87.7, 96.0)	97.6 (94.1, 99.4)	0.0 (0.0, 0.1)	97.6 (94.1, 99.4)
Uzbekistan	91.9 (88.4, 94.7)	6.4 (0.1, 29.3)	86.1 (65.3, 93.8)	92.5 (87.4, 95.7)	7.3 (0.1, 33.1)	85.8 (61.7, 94.9)
Vanuatu	88.6 (82.3, 93.1)	2.1 (0.0, 14.5)	86.7 (75.6, 92.1)	70.8 (56.8, 82.9)	3.3 (0.0, 21.0)	68.5 (51.8, 81.5)
Venezuela (Bolivarian Republic of)	69.4 (58.3, 78.9)	4.4 (0.0, 16.7)	66.3 (53.7, 77.2)	60.6 (47.9, 72.4)	7.5 (0.0, 30.9)	56.0 (39.4, 69.7)
Viet Nam	81.3 (74.0, 87.4)	1.8 (0.0, 12.2)	79.8 (70.4, 86.9)	90.2 (83.1, 95.1)	2.8 (0.0, 16.2)	87.6 (74.1, 94.4)
Yemen	75.1 (65.6, 83.1)	12.2 (11.8, 12.6)	65.9 (57.6, 72.9)	78.7 (67.2, 87.5)	28.7 (28.2, 29.2)	56.1 (48.1, 62.5)
Zambia	89.6 (83.7, 94.2)	0.2 (0.1, 0.5)	89.4 (83.6, 94.0)	91.6 (84.0, 96.5)	0.5 (0.3, 0.6)	91.1 (83.6, 96.0)
Zimbabwe	90.9 (85.9, 94.6)	5.1 (4.2, 6.1)	86.2 (81.2, 89.9)	89.6 (81.3, 95.0)	6.7 (5.8, 7.6)	83.6 (75.8, 88.6)

Expected coverage was expected levels for 2020 in the absence of the COVID-19 pandemic based on past trends. Estimated coverage reflects coverage for 2020 while accounting for estimated pandemic-related disruptions; more detail on these methods is in the main manuscript and appendix section 4. DTP3=diphtheria, tetanus, and pertussis, third dose. MCV1=measles-containing vaccine, first dose. UI=uncertainty interval.
